# Nutritional immunity: the impact of metals on lung immune cells and the airway microbiome during chronic respiratory disease

**DOI:** 10.1186/s12931-021-01722-y

**Published:** 2021-04-29

**Authors:** Claire Healy, Natalia Munoz-Wolf, Janné Strydom, Lynne Faherty, Niamh C. Williams, Sarah Kenny, Seamas C. Donnelly, Suzanne M. Cloonan

**Affiliations:** 1 School of Medicine, Trinity Biomedical Sciences Institute, Trinity College Dublin, Dublin, Ireland; 2 Tallaght University Hospital, Dublin, Ireland; 3 Joan and Sandford I. Weill Department of Medicine, Division of Pulmonary and Critical Care Medicine, Weill Cornell Medicine, New York City, USA

**Keywords:** Nutritional immunity, Microbiome, Immunity, Metals, Iron, COPD, Asthma, IPF, Mycobacteria

## Abstract

Nutritional immunity is the sequestration of bioavailable trace metals such as iron, zinc and copper by the host to limit pathogenicity by invading microorganisms. As one of the most conserved activities of the innate immune system, limiting the availability of free trace metals by cells of the immune system serves not only to conceal these vital nutrients from invading bacteria but also operates to tightly regulate host immune cell responses and function. In the setting of chronic lung disease, the regulation of trace metals by the host is often disrupted, leading to the altered availability of these nutrients to commensal and invading opportunistic pathogenic microbes. Similarly, alterations in the uptake, secretion, turnover and redox activity of these vitally important metals has significant repercussions for immune cell function including the response to and resolution of infection. This review will discuss the intricate role of nutritional immunity in host immune cells of the lung and how changes in this fundamental process as a result of chronic lung disease may alter the airway microbiome, disease progression and the response to infection.

## Background

Respiratory disease remains a global leading cause of death despite the advancements made by respiratory research [1]. Furthermore, those with poor respiratory health have a greatly diminished quality of life and recurrent hospitalizations. To alleviate the immense burden of respiratory disease we need a greater understanding of the resident and recruited immune cells in the lungs, their role in respiratory disease, and their complex interplay with the lung microbiome and with invading pathogenic microorganisms. Metabolite and nutrient availability in the lung, whether produced by the host or the microbiota, are likely to have a crucial role in the progression of lung disease as many immune cells require these specific nutrients to fuel their immune function when activated.

Nutritional immunity is classically defined as the sequestration of trace metals, most notably iron, by the host organism in an effort to block bacterial metal acquisition and thus limit disease progression during infection. Trace metals are essential to all forms of life. Most organisms require manganese, cobalt, iron, copper, nickel, selenium and zinc. Metal cofactors serve both catalytic and structural roles in a range of biological processes. With the exception of zinc, these metals are redox-active and this property contributes both to their catalytic activities and toxicity. Iron is the most abundant element on Earth and is also the most widely used metal in biological processes. This dependence on iron for most organisms makes it a vital currency in the context of host–pathogen interactions. The host takes advantage of this by sequestering iron during infection as a means of blocking iron acquisition by the invading microorganism, whereas bacteria have evolved mechanisms to steal iron from the host despite host iron sequestration [2]. Many respiratory pathogens such as *Mycobacterium tuberculosis*, *Pseudomonas aeruginosa*, *Klebsiella pneumoniae* and *Haemophilus influenzae* require iron and use several iron acquisition strategies [3–6]. Similarly, the commensals that comprise the microbiome require iron and other metals to support their metabolism. Thus, iron homeostasis and iron sequestration in the lung is crucial to controlling lung infections and to support a normal microbiome [7, 8]. Though nutritional immunity was first described and best characterized for iron, the sequestration, and intoxication of other metals including, zinc, manganese and copper has also been described [2, 9].

While the concept of nutritional immunity has traditionally encompassed the sequestration of metals from microbes, the innate and adaptive immune systems can also actively utilize metals to facilitate bactericidal function. For example, the host utilizes copper to exert a bactericidal effect through both redox-dependent and independent mechanisms limiting bacterial growth and facilitating bacterial death [10–12]. Similarly, the S100 family of calcium-binding host proteins, primarily calprotectin, are commonly found at the site of infection, where they can chelate free zinc, iron, nickel, copper and manganese and exert bactericidal activity in addition to nutrient deprivation having a pleiotropic role in the nutritional immune response [13]. Less ubiquitous metals also have a role to play: selenium, as part of selenoproteins, is important in defence against viral replication, and host manganese can reduce superoxide dismutase (SOD) activity in pathogens [14]. The proper storage, metabolism, and utilisation of these metals by immune cells is therefore critical both in facilitating immune cell function and depriving microbes of the necessary nutrients for survival and proliferation in the host. Intriguingly, pathogen reliance on the acquisition of host-derived metal supports further investigations into therapeutic avenues for metal chelators in chronic and acute lung infection.

Finally, metabolites created through metal-dependent metabolic pathways are also essential for the function of immune cells such as macrophages, neutrophils and T-cells [15]. As our understanding of the rapidly emerging field of immunometabolism expands, we are now beginning to appreciate that trace metal biology may also be essential for the correct functioning of key metabolic pathways (e.g., glycolysis, fatty acid oxidation, the tricarboxylic acid cycle, etc.) engaged by immune cells. We could therefore expand the meaning of nutritional immunity to encompass not just the battle over trace metals but also the role of all metabolites and nutrients that are dependent on metal biology important for host pathogen interactions.

This review will discuss the key roles for metals in lung immune cell function, how metals are altered and dysregulated in chronic respiratory disease and the evidence if any, for the role of metals in dictating the repertoire of commensal bacteria present in the lung as well as the role of nutritional immunity in the response of the lung to infection.

## Metals, innate immune cells of the lung and response to infection

Metal cofactors serve both catalytic and structural roles in a range of biological processes and play an important role in the development, maturation and function of immune cells. The role of metals in the biology of immune cells that are present in the lung is discussed below.

### Monocytes and macrophages

Macrophages are a heterogenous family of professional phagocytes and are the most abundant immune cell present in the lung under homeostatic conditions [16]. Their key role in the respiratory immune response is highlighted by macrophage dysfunction contributing to chronic lung diseases including chronic obstructive pulmonary disease (COPD) [17], asthma [18] and cystic fibrosis [19]. Their phagocytic abilities make them excellent surveillance cells both in the context of lung homeostasis and inflammation or infection. Tissue resident macrophages maintain immune homeostasis by carrying out essential housekeeping roles such as tissue repair while also acting as a first line of defence against microbial infections [20]. There are two major classes of lung resident macrophage populations. Alveolar macrophages (AMs) are the most abundant and are easily characterised for their low levels of the phagocytic receptor CD11b, their autofluorescent nature and high levels of the integrin CD11c and the lectin SiglecF [21]. AMs originate from foetal liver cells in the embryonic yolk sac where under the influence of granulocyte macrophage-colony stimulating factor (GM-CSF) they remain and sustain themselves within the alveoli [21, 22]. AMs reside within the lumen of the alveolus in close proximity to the alveolar epithelium and are directly exposed to air and the environment. AMs phagocytose antigens but also maintain homeostasis in the lung by catabolizing surfactant, removing particles as well as limiting inflammation [23, 24]. Originally thought to be sessile, AMs have recently been shown to be motile and to continuously crawl and cleanse all alveoli of particulate matter [25]. They also have the ability to communicate immunosuppressive signals to alveolar epithelial cells (AEC) [26]. AMs act as first line defence for respiratory pathogens, including bacteria and viruses such as *Streptococcus pneumonia*, *Mycobacterium tuberculosis* and *influenza* [27–29]. During infection AMs limit the inflammatory response by producing anti-inflammatory cytokines and by promoting tissue repair upon pathogen clearance [30, 31].

The second major resident lung macrophage population is the interstitial macrophage (IM) which is comprised of three phenotypically distinct subpopulations identified by their differential expression of CD11c, major histocompatibility complex (MHC)II and the mannose receptor CD206 [32, 33]. While their precise location in the lung remains controversial, IMs have been found in the lung parenchyma, both in the interstitium of the alveoli and the bronchovascular bundles with some specific subpopulations associated with nerves and blood vessels [32]. IMs originate and are maintained by circulating progenitor cells. They are smaller than AMs, immunoregulatory, and capable of antigen presentation [34].

In the setting of injury, inflammation, disease or with aging, resident AMs and IMs are often depleted and require the assistance of monocyte-derived macrophages that infiltrate the lung [35–38]. Once in the lung these infiltrating macrophages are defined by niche-derived tissue-instructive signals that trigger expression of specific differentiation programs, thus tailoring a particular lung specific functional identity [39].

#### Macrophages, iron and heme

Macrophages have been termed the “ferrostat” of tissue iron homeostasis [40]. Macrophages are vital for systemic iron homeostasis; supplying, sequestering or recycling iron as needed for erythropoiesis, bacteriostasis and erythrophagocytosis and constitute the main iron reservoir among immune cells and the third most important in the body after haemoglobin and liver ferritin stores [41]. Tissue-resident macrophages sequester and secrete iron on demand regulating local iron availability and modulating the tissue microenvironment, contributing to cellular and tissue function [40]. The exposure of AMs to a multitude of exogenous and endogenous sources in the lung, position AMs as key regulators of iron in the lung. As such, the storage, metabolism and detoxification of iron by AMs is paramount in their protection of alveoli against oxidative damage and maintenance of their innate immune functions (Table 1).

**Table 1:** Metal homeostasis within immune cells

	Iron	Zinc	Copper
Macrophages	DMT1 [42] Transferrin Receptor 1 (TFR1) [45] Slc39a14 (ZIP14) [77] Lactoferrin receptor (LfR) [43] CD163 [45] CD64 [220] Ferroportin (FPN) [50] Lipocalin-2 (LCN-2) [63] Nramp-1 [45] Hepcidin [53]	SLC39A (ZIP) Slc39a2 [77] Slc39a4 [77] Slc39a14 [77] SLC30A (ZnTs) Slc30a1 [77] Slc30a3 [77] Slc30a5 [77]	CTR1 [84] ATP7A [84]
Neutrophils	Nramp-1 [63] Lipocalin-2 [63] Myeloperoxidase [113] Ceruloplasmin [119] Lactoferrin [110]	Calprotectin (S100A8/S100A9 heterodimer) [13] Calgranulin C (S100A12) [124]	Calgranulin C (S100A12) [124] ATP7A [118] ATP7B [118] Ceruloplasmin [119]
NK cells	TfR1 (CD71) [183] Lactoferrin receptor (LfR) [185]	KIR receptor [188] Surface receptors with tyrosine phosphorylation sites [190]	Unknown
Dendritic cells	Unknown	ZIPs and ZNTs [102]	Unknown
Basophils	Unknown	Methallothioneins (MTs) [158]	Unknown
Eosinophils	Eosinophil peroxidase (EPX) [152]	Unknown	Unknown
Mast cells	Unknown	Unknown	Ctr2 [161]
ɣδ T cells	Lactoferrin receptor (LfR) [195] Hereditary hemochromatosis susceptibility gene (HFE) [197, 198] Β2-microglobulin (βm-2) [198]	Unknown	Unknown
iNKT cells	Ferroportin (FPN) [201] Hepcidin [201]	Unknown	Unknown
MAIT cells	Unknown	Unknown	Unknown
T cells	TfR1 (CD71) [209] Lactoferrin receptor (LFR) [195]	Unknown	Unknown
B cells	TfR1 (CD71) [209]	Unknown	Unknown

**Fig. 1:**
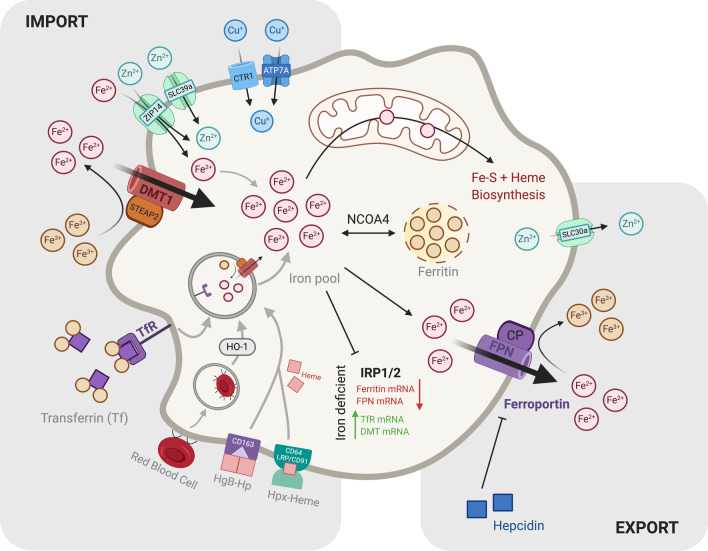
Macrophage Metal Metabolism. This brief summary of the metal metabolism of a generic macrophage highlights several mechanisms by which a macrophage can obtain metals. There are numerous metal transport proteins and receptors involved to ensure an adequate supply of these essential metals to the macrophage. (1) Macrophages obtain iron from various sources. Free circulating iron is first reduced from ferric to ferrous iron and then imported into the macrophage via protein transporters such as DMT1 and ZIP14. Transferrin-bound iron is bound to the transferrin receptor on the cell surface and imported via receptor-mediated endocytosis. Some macrophages also have the ability to obtain iron via erythrophagocytosis while others express receptors such as CD163 and CD64 allowing for the uptake of haemoglobin-haptoglobin and heme-hemopexin complexes, respectively. Once iron enters the macrophage it has numerous fates depending on the activation state of the cell. It can be stored in its ferrous state within the ferritin complex or utilized in the mitochondria for Fe-S cluster and heme biosynthesis. Iron is exported out of the macrophage through the only known iron export protein, ferroportin (FPN) whose expression is regulated by the hormone hepcidin. Further post-transcriptional regulation of intracellular iron levels is carried out by cytosolic regulatory proteins IRP1 and IRP2. (2) Intracellular zinc supply is mediated by two families of transport proteins; SLC39A (ZIPs) import zinc into the cell while SLC30A mediates zinc export out of the cell. (3) The copper importers Ctr1 and ATPase metal pump, ATP7A are two known copper transporters within the macrophage and gets upregulated to facilitate copper mediated host defence mechanisms

Following gut absorption of dietary and heme-conjugated iron, ferric iron (Fe^3+^) is bound to the glycoprotein transferrin for systemic circulation. Uptake of iron by AMs is mediated through the iron transporters transferrin receptor 1 (TFR1) and divalent metal transporter 1 (DMT1); AMs also express the additional iron uptake proteins including low-density lipoprotein receptor-related protein 1 (LRP1) and the zinc uptake receptor ZIP-14 (Fig. 1) [42–45]. Once inside the cell, iron dissociates from transferrin and is reduced to ferrous iron (Fe^2+^) for storage in ferritin, a ‘nanocage’ like structure for safe storage of iron within the cytoplasm [46]. The transmembrane protein ferroportin (FPN) exports ferrous iron from AMs into the extracellular space, where it is oxidised and bound to transferrin in serum [42]. FPN is the only known exporter of elemental iron, allowing the release of iron into the circulation and to other cell types. In the lungs FPN is highly expressed in epithelial cells and AMs [47, 48]. Decreased levels of FPN are observed in response to infection, in a bid to reduce cellular iron efflux and extracellular iron levels [49, 50]. The systemic iron regulator hepcidin, expressed in response to pro-inflammatory cytokines and bone morphogenic protein 6 signalling, inhibits FPN to reduce circulating iron (**Fig. ****1**) [51, 52]. While hepcidin does not seem to significantly contribute to AM lung iron trafficking [53], AMs produce endogenous hepcidin in response to challenge with the endotoxin lipopolysaccharide (LPS), potentially to sequester iron intracellularly through FPN degradation [53]. Indeed, this AM-produced hepcidin has proved essential for AM bactericidal function [54]*.*

Heme (iron-protoporphyrin IX) is an essential metallocofactor and signalling molecule across all cell types. While in highest demand in erythroid cells, heme synthesis is an evolutionarily conserved process occurring in virtually all eukaryotic cell types. De novo heme synthesis is a multienzyme process which starts and culminates in the mitochondria, with the first step of glycine and succinyl CoA condensation by 5-aminoevulinate synthase (ALAS) constituting the rate-limiting step in non-erythroid cells like AMs [55]. The hydrophobicity and cytotoxicity of heme necessitates carefully regulated handling in the cell, with such mechanisms of paramount importance in macrophages owing to their roles in erythrophagocytosis and heme degradation [56]. Unlike reticuloendothelial macrophages, which recycle iron from senescent erythrocytes via erythrophagocytosis [57] AMs in the alveoli do not seem to necessitate erythrophagocytosis at homeostasis. Instead, AMs uptake haemoglobin-haptoglobin and heme-hemopexin complexes via CD163 and CD63 respectively. However, AMs of patients exhibiting lung haemorrhage show iron overload, suggesting during increased heme burden AMs possess some erythophagocytic capability [58]. AMs play important roles regulating the iron pool by sensing free heme (the iron-containing porphyrin, key to O_2_ transport and storage among other biological processes) at concentrations reflective of pulmonary haemorrhage. AMs utilize heme to produce reactive oxygen species (ROS) and nitric oxide (NO) enhancing their bactericidal and phagocytic capabilities [59]. Importantly, erythrophagocytosis also upregulates the expression of heme oxygenase 1 (HO-1) in macrophages leading to heme degradation. Heme catabolism by HO-1 exerts anti-inflammatory effects through the products of heme degradation: bilirubin (arising from the reduction of biliverdin), carbon dioxide and ferritin all of which possess anti-inflammatory activity [60–62]. This illustrates the importance of iron metabolism, particularly of the HO-1/heme axis in maintaining AM function and lung homeostasis.

**Fig. 2:**
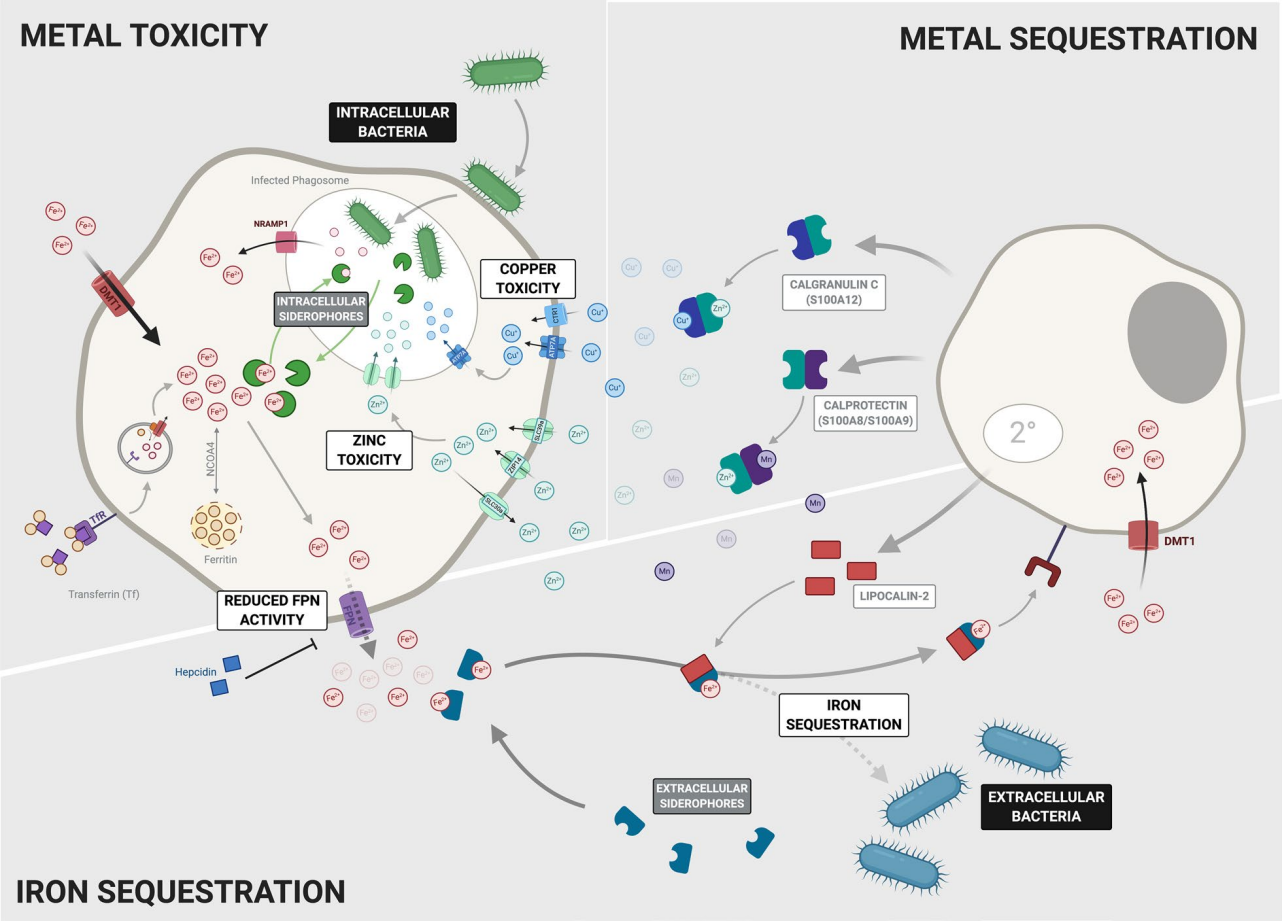
Host Bactericidal Immune Defence Facilitated by Trace Metals. Metals play a pivotal role in contributing to the host immune response during bacterial infection. They can be used by immune cells to either mediate metal toxicity to rapidly kill the bacteria or be sequestered away in order to deprive the bacteria of essential metals in order to limit bacterial growth and replication. Whether the metals are sequestered or used to facilitate toxicity mechanisms is highly dependent on the nature of the bacterial infection. (1) Intracellular bacteria are mainly located within the phagosome of their host cell. Here they release siderophores allowing them to scavenge iron within the cell. In response, the host limits bacterial iron supply by exporting iron out of the infected phagosome via the NRAMP1 transporter. Furthermore, the host employs toxicity mechanisms by which both zinc and copper are pumped into the infected phagosome via their respective transporters in order to limit bacterial growth and replication. (2) Extracellular bacteria produce numerous different proteins to mediate metal acquisition from the host. The majority produce siderophores, which vary between bacterial species. However, they all function to supply the bacteria with an adequate amount of iron. The host also employs mechanisms to limit the amount of metals available to the bacteria in the extracellular space. Iron is sequestered by the upregulation of hepcidin resulting in the degradation of FPN which reduces the amount of iron exported from the cell. Furthermore, the iron importer DMT1 is also upregulated in both macrophages and neutrophils. Several metal binding proteins have also been shown to be produced mainly by neutrophils to bind extracellular metals. Lipocalin2, produced in secondary granules of neutrophils, binds bacterial siderophores and shuttles them away from the bacteria to the host cell. S100 proteins such as Calprotectin that binds zinc and manganese, and Calgranulin C binding zinc and copper also sequester these metals out of the extracellular space

Iron-sequestration is of particular importance to limit pathogen outgrowth in the lungs. AMs act as the first line of defence against respiratory pathogens and contribute to limiting iron availability to pathogens. An example is the ability of AMs to secrete the siderophore lipocalin-2 (LCN-2) during infection that binds to enterobactin-type and mycobacterial siderophores to sequester free iron (Fig. 2) [63]. Macrophage polarisation also differentially regulates iron-response in AMs. Iron accumulation in M1-polarized AMs promotes a bacteriostatic response to the anaemia of chronic infection and simulates expression of pro-inflammatory cytokines, potentially through hepcidin-mediated FPN downregulation [64]. In contrast, M2 cells favour iron release linked to upregulation of FPN a phenotype that may be driven by upregulation of HO-1 [65]. Reciprocally, iron can also modulate monocyte polarization. In vivo murine models of iron overload drive macrophage polarization to favour the M2 phenotype mitigating pro-inflammatory responses [66]. However, in vitro studies of macrophages isolated from chronic venous leg ulcers showed excessive erythrophagocytosis and an ensuing high intracellular iron concentration to promote M1 polarization [67]. Likely, iron concentration is crucial in modulating polarisation – the tissue iron deposition exhibited in venous leg ulcers is extremely high, whilst the dietary iron supplementation approach used in the murine studies provides a moderate dose [66]. While the effects of iron overload in the lung on macrophage polarization remain unclear, it will be interesting to see if such a concentration-dependent mechanism is also observed.

Macrophages carry out a vast array of effector functions and must be able to adopt several activation states by changing their metabolism to fuel these functions [68]. The influence of macrophage immunometabolism in determining an inflammatory or anti-inflammatory phenotype have been extensively reviewed [69]. However little attention has been given to the role that metals play in the regulation of immunometabolic networks. Iron-sulfur (Fe-S) clusters play an essential role in macrophage metabolism by acting as cofactors to essential metabolic proteins and mediating the electron transport chain. For example, the mitochondrial enzyme lipoic acid synthase (LIAS) requires Fe-S clusters to aid in the synthesis of lipoamide-requiring enzymes such as pyruvate dehydrogenase (PDH) and α-ketoglutarate dehydrogenase (α-KGDH). Furthermore, the proteins involved in electron transport, such as ferredoxins also require Fe-S clusters as a part of their redox-active centres to ultimately produce ATP [70]. In response to inflammatory stimuli Fe-S clusters are also regulated. LPS/interferon-ɣ (IFN-γ) stimulation of murine bone marrow derived macrophages (BMDMs) and RAW264.7 macrophages downregulates two key Fe-S cluster biosynthesis proteins (cysteine desulfurase Nfs1 and its partner IscU) possibly to avoid damage to Fe-S clusters by NO [71]. In vivo stimulation with *Mycobacterium bovis* Bacille Calmette Guerin (BCG) or in vitro stimulation with IFN-γ inhibits aconitase activity in macrophages by removing an iron atom for the Fe-S prosthetic group of aconitase which is essential for its catalytic activity in the TCA cycle. This inhibition of aconitase coincides with the downregulation of mitochondrial respiration upon its activation and also a compensatory increase of glycolysis [72]. Similarly, acute iron deprivation of primary human macrophages enhances glycolysis by upregulating hypoxia-inducible factor 1-α (HIF-1α) regulated genes while downregulating oxidative phosphorylation (OX PHOS) via the iron-responsive transcription factor ATF-4. Furthermore, when these iron-deprived cells were stimulated with LPS their proinflammatory functions were impaired [73].

#### Macrophages, zinc and copper

Like iron, zinc is an essential cofactor for life. Vertebrates have evolved complex transport and buffering systems to maintain zinc homeostasis. The balance of zinc homeostasis is crucial during infection to block access to zinc from invading microorganisms, but also to ensure the function of immune cells. Zinc deficiency in humans is associated with impaired response to several infectious diseases [74, 75]. Zinc deficiency leads to reduced phagocytic ability of macrophages, while zinc supplementation can improve phagocytosis but the molecular mechanisms behind how zinc affects this process is not yet known [74–76]. Cellular zinc homeostasis is regulated by two families of zinc transporters, the SLC39A importers (ZIPs) and the SLC30A exporters (ZnTs). Zinc plays an important role in macrophage efferocytosis involving the coordinated action of the ZIP1 and ZIP2 importers [77]. The roles of other zinc transporters in different infection contexts is not yet known but the exporter SLC39A10 is vital for mediating zinc homeostasis in macrophages in response to LPS stimulation resulting in increased macrophage survival (**Fig. ****2**) [78]. *Slc30a1 (ZnT1)* is induced in human macrophages during *M. tuberculosis* infection and zinc toxicity is a strategy used by *M. tuberculosis* infected macrophages [79]. Zinc accumulation was also observed in phagosomes containing *E. coli* contributing to its killing, suggesting that this may be a general antimicrobial strategy employed by macrophages [80].

Copper is used as an essential cofactor in an array of biological processes in many forms of life, including microorganisms. Copper deficiency increases susceptibility to many types of infections indicating an important role for this metal in host defence [10, 81, 82]. Furthermore, the prevalence of copper resistance mechanisms in pathogenic microbes highlights the importance of copper toxicity as a defence strategy in mammals. Macrophages exploit both the essentiality and toxicity of copper to defend against microbes. As a host defence strategy, copper levels rise within phagosomes of peritoneal macrophages upon infection with mycobacteria [83]. The copper importer Ctr1 and the ATPase copper pump ATP7A are induced by IFN-ɣ in macrophages. The ATPase pump is trafficked to phagosome compartments and is required for the bactericidal activity of macrophages (Fig. 2) [84]. Copper toxicity is a host defence strategy against other respiratory pathogens such as *S. pneumoniae* as pulmonary macrophages were shown to be more efficient at clearing pneumococcal bacteria lacking the CopA copper efflux pump [85]. Conversely, IFN-ɣ activated macrophages reduce copper levels in phagosomes to control the fungal pathogen *Histoplamsa capsulatum* [86]. Fungi have high tolerance to copper and so copper toxicity as a defence strategy is not suitable for these micro-organisms, thus macrophages restrict this essential nutrient from fungi within the phagosome.

#### Macrophages and other trace metals

The role of other trace metals in macrophage biology is poorly studied and has mainly focused deciphering additional metal binding capabilities of proteins implicated in iron biology. In addition to iron, the membrane transporter NRAMP1 also has affinity for manganese, transporting it across the phagosomal membrane [87]. Transferrin has been shown to bind manganese in vitro and is the main manganese-positive protein in circulation in vitro, although whether macrophages can take up manganese-laden transferrin through TFR1 remains to be defined [88].

While numerous selenoproteins key in cellular processes have been characterised, their precise roles in processes governing the macrophage immune response are poorly defined. Selenoprotein methionine sulfoxide reductase B1 (MsrB1) expression is induced in macrophages upon LPS stimulation, where it regulates actin assembly and production of anti-inflammatory cytokines [89]. This anti-inflammatory role is concordant with observations that dietary selenium supplementation drives macrophages towards the alternatively activated M2 phenotype in an IL-4 dependent manner [90]. Macrophages deficient in the selenocysteine tRNA showed diminished migration in vitro*,* but no change in inflammatory response [91]. Targeting of specific selenoproteins in future studies will allow a greater understanding of the intricacies of selenium regulation in macrophages.

### Dendritic cells

Dendritic cells (DCs) play a crucial role in the initiation and regulation of the immune response by functioning as powerful antigen presenting cells. DCs resident in the lungs can be divided into three subsets that are of independent origin and have distinct functions depending on their location [92]. Regardless of their subtype, DCs develop in the bone marrow from hematopoietic stem cells into early progenitor cells where they then branch off into their specific subsets [93]. Conventional DCs (cDCs) in the lung express either CD103 or CD11b. CD103^ +^ DCs (cDC1) are located in the respiratory epithelium where they sample contents of the conducting airways by projecting their long dendrites between epithelial cells into the airway lumen. In contrast, CD11b ^+^ DCs (cDC2) are lodged under the basement membrane of the lamina propria [94]. Once the cDCs sample an antigen, they migrate the draining lymph nodes where they present the processed antigen to naïve T cells which drives specific T cell polarization and proliferation [95].

Monocyte-derived DCs (moDC) are recruited to the lung during infection or inflammation to help drive the immune response. The existence of these DCs remains controversial as they express markers such as CD11c, CD64 and the MAR-1 antibody which can also represent a macrophage cell population [94, 96]. Plasmacytoid DCs (pDC) secrete large amounts of type I interferon (IFN) during viral infections and have been located in the lymphoid follicles of the small airways [97, 98]. Furthermore, pDCs also play an immunoregulatory role by sensitising T helper (Th) type-2 cells to harmless antigens in the lung while suppressing the generation of effector T cells. However, this tolerance is suggested to be suppressed when the pDCs interact with a virus and produce IFNs [99].

#### Dendritic cells and metals

Not much is known about the role that metals may play in the function of dendritic cells. Iron levels affect the development of DCs from bone marrow progenitors whereby high iron conditions used to culture bone marrow cells leads to a defective development of moDCs that are unable to respond efficiently to LPS [100]. On the other hand, iron deficiency in vitro also leads to the improperly differentiated moDCs that are unable to stimulate T cells [101]. Interestingly, reduced intracellular free iron levels promote the activation of DCs. Stimulation of DCs with LPS leads to an NFκB dependent increase of ferritin.

Zinc has also been implicated in DCs response to stimulation. A study identified a link between toll like receptor (TLR) signalling and zinc homeostasis in these immune cells. Stimulation of DCs with the TLR4 ligand LPS lead to altered expression of zinc transporters (both importers and exporters), and a decrease in intracellular free zinc [102]. Treating DCs with a zinc chelator resulted in the same effects as LPS stimulation, in terms of upregulation of MHC class II and costimulatory molecules. Further investigations are needed to understand the link between DC zinc homeostasis and their response to TLR stimulation.

### Neutrophils

Neutrophils are short-lived, highly mobile phagocytes that constitute hallmarks of acute infection. As granulocytes neutrophils have enzyme-filled granules that they use to fight off infections in addition to their phagocytic capabilities and synthesis of chromatin-derived neutrophil extracellular traps (NETs) [103]. Despite their prominent phagocytic role in the response to acute infection, neutrophil function is remarkably plastic, with secreted effectors driving pro- and anti-inflammatory neutrophil function [104–106]. Neutrophils also possess the capacity to modulate the functionality of other immune cells through secreted effector proteins, with the cleavage of TLRs and cytokines by neutrophil elastase associated with altered macrophage function [107]. Given its frequent exposure to environmental pathogens, it is no surprise that the lung is a major neutrophil reservoir. Studies in a rabbit model have shown the upregulation of P-selectin by AECs in response to internal and external stimuli binds P-selectin glycoprotein ligand-1 on circulating neutrophils, facilitating further binding and subsequently tissue extravasation [108]. The majority of marginated neutrophils in the lung reside in the capillary bed, expressing the chemokine receptor (CXCR) 4 to promote their retention [109]. Such margination in the microvasculature positions a large pool to kill inhaled bacterial, fungal and viral pathogens through phagocytosis, NETosis and release of antimicrobial cytokines [107].

#### Neutrophils and iron

Iron is central to numerous neutrophil inflammatory responses. Neutrophils secrete LCN2 from their secondary granules to bind bacterial siderophores and limit their iron acquisition, and express NRAMP1 to sequester free iron from serum during infection [63]. Studies in the iron transport protein lactotransferrin (lactoferrin)-deficient mice have illustrated a role of neutrophil-secreted lactoferrin in modulating the oxidative burst response [110]. Iron-unsaturated lactoferrin was shown to inhibit human neutrophil apoptosis in vitro, suggesting a role in chronic neutrophilic inflammation [111]. Binding of recombinant human lactoferrin to human neutrophils in vitro induces neutrophil activation although the precise mechanisms of lactoferrin binding to the neutrophil surface remain uncharacterised [112]. Neutrophils also express the heme-containing myeloperoxidase, which catalyses the generation of hypochlorite anions to drive degranulation and NETosis [113].

Iron availability may also influence metabolic reprogramming in neutrophils. HIF-1α is regulated by a family of prolyl hydroxylases (PHDs) and under normoxic conditions the PHDs rapidly hydroxylate HIF-1α and so target it for degradation [114]. *Phd2*^*−/−*^ neutrophils have higher ATP levels, increased abundance of pentose phosphate pathway intermediates, more glycogen stores and a greater glycolytic capacity [115]. This suggests that neutrophils, like macrophages, require HIF-1α to engage in glycolysis. Low oxygen tension is not the only way to block HIF-1α hydroxylation, as PHDs also require α-ketoglutarate, Fe^2+^ and ascorbate. Increased expression of HIF-2α, regulated in a similar manner to HIF-1α, has been linked to decreased death of neutrophils by apoptosis, and iron chelators have been shown to inhibit apoptosis in neutrophils [116]. Heme metabolism has also been implicated in neutrophil function during respiratory infections as HO-1 expression in the lung is also associated with reduced neutrophil recruitment in murine models of LPS-induced pulmonary inflammation [117].

#### Neutrophils and other metals

Copper is required for proper NETosis, with mice carrying mutations in the copper transporters ATP7A and ATP7B showing a reduced capacity for NET release during systemic inflammation [118]. Ceruloplasmin, a Cu^2+^ dependent ferroxidase elevated in serum during acute infection, is secreted by human neutrophils to upregulate ROS in a potentially bactericidal mechanism [119]. Calprotectin is released from the cell during degranulation as part of NETs, where it is essential for antifungal activity [13].

Neutrophil expression of protein effectors binding other trace metals is crucial in their bactericidal function. Zinc deficiency induces expression of the zinc-binding heterodimer calprotectin and detrimentally impacts neutrophil phagocytic capacity through impairing ROS function, along with oxidative burst, degranulation and cytokine production [120]. Indeed, zinc signalling has been shown to be vital in NET formation by neutrophils and granulocytes: both zinc excess and deficiency inhibit superoxide production in neutrophils, a prerequisite for NET formation [121]. Neutrophils express calprotectin which can bind zinc and manganese*,* attenuating the effects of zinc-associated enzymes such as matrix metalloproteases and manganese-dependent bacterial superoxide defences [122, 123]*.* Neutrophils also express calgranulin C (S100A12), which can bind copper and zinc to exert an antimicrobial effect and trigger monocyte recruitment [124]. In addition to iron and zinc, calprotectin can also chelate manganese in a manner that both sequesters the nutrient from pathogens and facilitates antimicrobial activity, with recombinant calprotectin defective in manganese binding unable to exert antifungal activity against *Aspergillus fumigatus* [125]. Indeed, calprotectin-mediated manganese and zinc sequestration is a host immune strategy against the *S. aureus* superoxide defence, enhancing the susceptibility of the bacterium to neutrophil killing [122] Little is known about the role of selenium in neutrophil function, although increased dietary intake may protect neutrophils from oxidative damage [126].

### Other granulocytes

#### Mast cells

Mast cells are granulocytic leukocytes that mediate inflammation through degranulation in response to infection, allergens and tissue injury [127]. Mast cells are unique among leukocytes in that they are released from the bone marrow as CD34 ^+^ mast cell progenitors and do not terminally differentiate until their recruitment to tissue [128]. These immature progenitors are minimally granulated and reach maturation upon tissue homing, where stem cell factor and other tissue-specific factors drive MCs towards a pro-inflammatory, anti-inflammatory or an immunosuppressive phenotype [129]. Mast cell activation is regulated by several cell surface receptors, including TLRs, cytokine and chemokine receptors, hormone receptors and FcεRI and Fcγ receptors. The ensuing combination of stimuli facilitates a pleitropic role for mast cells in the immune response and leads to the secretion of a wide range of effector proteins: preformed mediators rapidly released upon activation (TNF-α, histamine and proteoglycans), and mediators synthesised following activation (lipid mediators, growth factors, cytokines and chemokines) [130–132]. Mast cell frequency in the lung at homeostasis is low and interspecies variation in mast cell populations have made the homing process difficult to study, although the identification of a multitude of mast cell chemoattractants means it likely occurs through a regulated recruitment process [133, 134].

#### Basophils

Basophils similar to mast cells contain granules within their cytoplasm [135], express the high affinity receptor for IgE (FcεRI) and release proinflammatory mediators such as histamine when this receptor becomes cross-linked. Both are derived from CD34 ^+^ haematopoietic stem cells located in the bone marrow [136]*.* Despite their similarities, basophils and mast cells are morphologically and biochemically distinct cell types [137]. The type 2 immune response is initiated by the immune system when in contact with parasites which increases IgE production and the number of eosinophils, basophils and CD4 T cells [138]. Basophils drive this type 2 response by producing the cytokine IL-4 [139] but also plays a role in allergic reaction and airway inflammation. Basophils have quite a short lifespan and so they must be constantly replenished by the bone marrow. For many years it was accepted that basophils are present in circulation and only migrate to tissue in response to certain inflammatory signals [140]. However, murine studies have proposed the idea of tissue-resident basophils after it was discovered that basophils seem to be present at all stages of lung development [141]. Furthermore, these lung-resident basophils differ phenotypically from peripheral blood basophils and are driven by IL-33 and GM-CSF produced in the lung microenvironment. Localised in the alveoli, intriguingly basophils also seem to be responsible for guiding the development and function of AMs [141].

#### Eosinophils

Eosinophils are bone marrow-derived, granule containing, pro-inflammatory leukocytes. Terminally differentiated in circulation, eosinophils act as effectors in host defence from microbial and metazoan infection and allergic reaction, primarily through the activity of cationic proteins resident in the eosinophilic granule. Eosinophils comprise a mere 1–3% of circulating leukocytes and readily migrate to the lung during inflammation. Following their IL-5-induced proliferation and mobilization in the bone marrow, eosinophil recruitment into the lung is driven by local expression of the chemotactic factor eotaxin [142]. Lung-resident eosinophils reside in the parenchyma and display a marker expression pattern (Siglec-F^mid^CD62L^+^CD101^low^) distinct from those recruited to the airways (Siglec-F^hi^CD62L^−^CD101^hi^) although functional differences between the two subsets remain uncharacterised [143]. Murine models of chronic allergic asthma have shown secretion of CCL17 and CCL22 by dendritic cells recruits circulating eosinophils to the lung in response to allergic inflammation, where eosinophils recruit further DCs and Th2 cells [144, 145]. During viral respiratory infection, expression of TLR7 allows eosinophils to detect pathogen associated molecular patterns (PAMPs) and exert direct antiviral effects through degranulation and cytokine and superoxide production [146].

#### Metals and granulocyte function

There are many reports correlating altered iron metabolism with allergy and atopic airway hyperreactivity. There is a higher prevalence of allergic diseases in those with diseases causing high systemic iron levels [147]. As basophils, eosinophils and mast cells play a role in allergy, the altered iron homeostasis likely impacts the function of these cells, contributing to pathology. Reducing iron levels, by administration of an iron chelator reduces IgE serum levels in a rat model of Th2 mediated autoimmunity [148]. Iron supplementation results in a significant decrease of airway eosinophila in an ovalbumin-driven allergic asthma mouse model [149]. Neutrophils utilize the metalloprotein myeloperoxidase (MPO), which is a hemeprotein that plays a role host defence via oxidation [150]. Similarly, eosinophils have eosinophil peroxidase (EPX), which is a two-chain hemeprotein and has been shown to have homology to MPO in neutrophils [151]. Efficient iron stores and uptake is said to be required for the generation of EPX suggesting iron levels are important for eosinophil function and activity [152].

HIF-1α, whose activation is iron dependent was shown to accumulate after basophils were stimulated with anti-IgE [153]. Accumulation of HIF-1α is suggested to be linked to the reaction of basophils to the hypoxic environment and stress-induced conditions. Whether intracellular iron levels in basophils affects HIF1α and cell activation as it does in macrophages and dendritic cells remains to be determined. This would be an interesting avenue to explore as altered iron levels are linked with allergic inflammation.

Reduced zinc levels are associated with several allergic diseases including bronchial asthma and chronic rhinosinusitis [154–156]. Zinc is required in mast cells for degranulation and the production of cytokines and mast cell granules are reported to contain high levels of zinc [157]. FcɛRI is expressed on the surface of mast cells and basophils, and its activation leads to the release of cytokines (including IL-4) and chemical mediators. The requirement of zinc in basophil function has not yet been fully elucidated however the regulation of intracellular zinc levels by metallothioneins (MTs) has an important role in FcɛRI-driven calcineurin/nuclear factor of T cell signalling and IL-4 signalling in basophils [158]. Zinc suppresses eosinophilic inflammation, increased numbers of eosinophils in bronchoalveolar lavage fluid fluid (BALF) are observed in zinc deficiency while zinc supplementation reduces the numbers of eosinophils in BALF, similar to what is observed with iron [159, 160]. However, the molecular mechanism driving this zinc-associated eosinophil recruitment to the lungs is yet to be elucidated. While copper has been implicated in mast cell maturation, little remains known about the role of copper and other metals in granulocyte function [161].

### Innate lymphocytes

In addition to the innate and adaptive defences, the lung harbours innate-like lymphocytes types that constitute the second and third tiers of defence. Lung tissue-resident innate-lymphocytes include innate lymphocyte cells (ILCs) which include NK cells, invariant natural killer cells (iNKT), MR1-restricted T (MAIT) cells and γδT cells.

Natural killer (NK) cells are short-lived innate effectors that belong to type 1 innate lymphoid cells, and account for 5–25% of total CD45^+^Lin^−^ cells in the lungs [162, 163]. NK cell activation is mediated through expression of inhibitory and activating receptors. Inhibitory receptors (killer cell-immunoglobulin like receptor (KIR), CD94:NKG2A, ILT-2) recognise host major histocompatibility complex class I molecules to limit cytotoxicity towards the host and facilitate self-tolerance in the ‘missing self’ response [164]. Activating receptors (TLRs, NGK2D) recognise increased ligand expression on target cells to mediate the ‘induced self’ response [165]. NK cells migrate from the bone marrow to the lung. In humans, NK cells are found in the lung parenchyma whereas in mice they have been found in the alveoli during influenza infection [166, 167]. In the human lung NK cells constitute several heterogenous populations. CD16^+^ CD56^dim^ cells constitute 80% of lung NK cells and are a hyporesponsive, mature, cytotoxic population that express KIR [168]. Whether the lung contains a tissue resident population of NK cells has been debated. Marquardt et al. showed lung NK cells lack expression of the CD69 tissue resident marker; however, Yamamoto et al. showed that blocking NK cell recruitment has no effect on NK ability to control lung tumour growth, suggesting lung-resident NK cells are essential in controlling metastasis [167, 169]. Parabiotic mouse studies have shown this lung-resident population to be low relative to other tissues with a specific tissue and functional signal, potentially contributing to barrier functions in the lung [170]. Murine lung NK cells also have diminished proliferative capacity and cytotoxic activity than splenic NK cells [171], suggesting that the lung microenvironment may inhibit NK cytotoxic potential to prevent airway damage. NK cells play important roles in response to infection and cancer. Murine models have illustrated their importance against respiratory viruses, bacteria and fungi [172]. Their precise contribution during human lung infections is unclear, but they are important in the early response to influenza [173]. Loss-of-function mutations leading to NK cell deficiencies uncovered an increased susceptibility to recurrent bacterial respiratory infections [174]. NK cells have also been implicated in asthma [172] and COPD [172].

ɣδ T cells are a subset of unconventional or innate-like T cells that are characterized by the expression of a γ and δ chain T cell receptor (TCR) which require MHC for antigen recognition. ɣδ T cells are abundant in mucosal surfaces including the lungs [175] where they swiftly react to conserved non-peptide antigens and produce large amounts of cytokines. In the lungs, ɣδ T can constitute up to 20% of resident lymphocytes and contribute to maintain tissue homeostasis and fight infections and cancer. These cells play important roles against bacterial pathogens including *S. pneumoniae, B. pertussis, M. tuberculosis,* viruses i.e., influenza, and some fungi. Likewise, they promote protection in models of cancer. However, these cells can also contribute to pathology in asthma and lung fibrosis [176].

In addition to NK cells, ILCs include a heterogeneous population of other innate lymphocytes described both in mice and humans. These include lymphoid tissue inducer (LTi) cells and three subsets of ‘helper’ ILCs (ILC1, ILC2 and ILC3) which unlike NK cells, lack cytotoxic properties and secrete higher amounts of effector cytokines. Lung helper ILCs have been extensively reviewed [177]; these cells are present across all different parts of the respiratory tract and lungs, including upper respiratory tract, lung parenchyma and bronchoalveolar spaces, where they contribute to homeostasis, pathogen clearance and also pathology [177].

Invariant natural killer cells (iNKT) and MR1-restricted T (MAIT) cells are “innate-like” unconventional T cells that reside in the lung and other mucosal surfaces and play important roles during infection by recognizing non-peptidic antigens [178]. iNKT cells are αβ T cells which recognize glycosphingolipids and other microbial-derived glycolipids presented in CD1d molecules. iNKT cells can swiftly produce a range of inflammatory cytokines when activated and type I iNKT also present cytolytic activity against cancerous and virally infected cells [178, 179]. Lung type I NKT cells are mainly resident within the parenchyma (NKT17, which produce IL-17) or in the vasculature (NKT1 and NKT2) [180, 181]. MAIT cells recognize microbial-derived metabolites [178] and produce cytokines including IFN-ɣ, TNF-α, IL-17A, and IL-22 aiding in bacterial killing [182]. Their role and precise localization in the lung in homeostasis remains to be determined.

### Metals and innate lymphocytes

The influence of metals on innate lymphocytes has been less studied and seem to vary among different innate lymphocytes subsets.

#### NK cells and Iron

In contrast to monocytes and polymorphonuclear cells, little is known about a potential role for an iron-related nutritional immune response in NK cell function, although recent studies suggest a role for iron signalling pathways in NK cell activation. Specifically, expression of the transferrin receptor CD71 is upregulated in the NK cell surface during their maturation in the murine bone marrow and during activation with poly(I:C) [183]. Basal expression of CD71 in human NK cells at homeostasis is low and increases in response to cytokine treatment in vitro*,* although how this potential to increase iron uptake drives the activated NK cell phenotype is unclear [184]. Lactoferrin increases NK cell cytotoxicity in vitro*,* which could imply an antimicrobial role for NK cells in infection in response to neutrophil and macrophage secretion of lactoferrin [185]. NK cells of patients exhibiting systemic iron overload in myelodysplastic syndromes displayed upregulated c-Jun N-terminal kinase (JNK) and downregulated p38 expression, suggesting a role for iron in NK signal transduction pathways [186]. It is interesting to note that perturbed iron homeostasis in NK target cells affects NK cell recognition—iron depletion and ferritin heavy chain (FTH) in primary cancer cells was observed to increase NK cell targeting [187].

#### NK cells and other metals

Zinc signalling has also been suggested as a mediator of the NK cell phenotype. Recognition of surface MHC-1 on endogenous cells by the NK cell KIR is essential in for NK cell-mediated killing. Zinc is essential for KIR multimerization to form this ‘NK cell synapse’ [188]. Zinc supplementation in vitro stimulates the differentiation of human-derived CD34^+^ progenitors to NK cells [189]. NK cells also possess several surface receptors with tyrosine phosphorylation sites, suggesting a role for zinc in signal transduction; the cytotoxic capabilities of NK cells are reduced in zinc deficiency and increased in zinc excess, with the precise signalling pathways driving such a change remaining unclear [190]. Roles for other trace metals such as copper or manganese in NK cell biology are difficult to decipher; the few studies attempting to ascertain this were largely performed in vitro and thus may not hold significant physiological relevance [191]. Manganese supplementation promotes NK cell anti-tumoral activity in vivo [192]. Manganese also activated the adhesion protein lymphocyte function-associated antigen (LFA)-1, essential for NK cell cytotoxicity [191]. In vitro supplementation with selenium increases splenic NK cell cytotoxicity [193]. Further in vivo studies are required to ascertain the exact role of manganese and selenium on NK cell function and the biological pathways implicated.

#### iNKT, MAIT, γδT cells and metals

Limited data on ɣδ T cells suggest that variations in iron availability may not affect these cells as much as they affect their adaptive lymphocyte counterparts. ɣδ T cell express the TFR1 (or CD71) which is required by αβ CD4^+^ and CD8^+^ T cells during proliferation. While blocking TFR1 with an anti-CD71 antibody blocks proliferation of T cells, ɣδ T cells proliferation is not affected, suggesting these cells are either less dependent on iron or rely on other mechanisms for obtaining iron [194]. Indeed, activated γδ T cells express high levels of the lactoferrin receptor (LfR). Like transferrin, lactoferrin can bind 2 ferric ions and provide LfR-expressing cells of an iron source; in addition lactoferrin possesses antimicrobial activity [195]. Unlike transferrin, which is present at high concentrations in plasma, lactoferrin is abundant in secretions like breast milk, and fluids covering the linings of mucosae like saliva, mucus and BALF [195, 196]. Addition of lactoferrin to in vitro cultures increase the proliferation of γδ T cells upon mitogen stimulation. Given the important role of γδ T cells in defence against mucosal pathogens, high expression of LfR may be an adaptation for acquisition of lactoferrin-bound iron in mucosal sites including the lungs.

A functional link between the hereditary hemochromatosis (HH) susceptibility gene HFE (also known as homeostatic iron regulator), iron status and adaptive T cell function has been suggested [197]. However, if or how HFE regulates ɣδ T cells remains unknown. HFE encodes a non-classical MHC molecule; as such the protein may have the ability to form complexes with β2-microglobulin (β2-m) [198]. Interestingly, mice deficient in β2-m display iron overload patterns similar to HH [199] and have enhanced homeostatic proliferation of ɣδ T cells. Since HFE deficiency is linked to iron overload, it is possible to hypothesize that excess iron could be linked to the altered ɣδ T cell proliferation in these mice and maybe also in HH patients. In addition to the altered lymphocyte ratios, some HH patients may have altered iNKT numbers. Iron overload is associated with reduced numbers of iNKT cells, which was more notable in untreated patients [200]. Intriguingly, the regulation of iron levels and iNKT activation/proliferation seems to be two-way: iron levels affect iNKT numbers, and iNKT cells can affect iron homeostasis. In vivo*,* activation of mouse iNKT cells by injection of their prototypical antigen α-galactosylceramide (α-GalCer), not only induces iNKT proliferation but also promotes early hepcidin expression while suppressing FPN. Activation of iNKT cells also lead to an early peak of serum iron followed by accumulation of iron in the spleen and liver. These effects were abolished in *Jα18*^*−/−*^ mice which particularly lack iNKT cells. This indicates that iron and iNKT cells reciprocally regulate each other [201]. More research is needed to understand the effects of iron and other metals on MAIT cells and other groups of innate lymphocytes.

## Metals and adaptive immune cells of the lung

### Adaptive lymphocytes (B and T cells)

T and B cells are at the centre of cellular adaptive immune responses. These cells are virtually capable to recognize infinite antigens in a highly specific manner thanks to their TCR and B cell receptors (BCR) and offer long-term protection against infection thanks to the generation of effector and tissue resident memory T lymphocytes and long-lived antibody producing plasma cells. Adaptive lymphocytes can be found in different lungs compartments. Conventional CD4^+^ and CD8^+^ T lymphocytes populate the mucociliary epithelium of the conducting airways in the trachea and bronchi. In contrast, B cells (mostly IgA^+^) and most CD4^+^ T cells are found in the bronchial lamina propria. Early studies also reported the presence of lymphocytes in the lung interstitium in similar numbers to those found in the circulation [202]. In addition, an intravascular pool has been identified. T CD4^+^, T CD8^+^ and B lymphocytes have been isolated from the alveolar spaces. B cells constitute only 5–10% of the total lymphocytes isolated in BALF [202].

T and B lymphocytes can also accumulate in tertiary lymphoid structures (TLS) in the lungs. Bronchial Associated Lymphoid Tissue (iBALT) are the main TLS in the lung and serve as priming structures for B and T cells. iBALT organization is similar to secondary lymphoid organs, presenting a T cell zone and a B cell follicle. These structures are readily detectable in children but only inducible and transient in adults. Once iBALT is formed, it can be maintained in the lungs for months even in the absence of the original stimulus that triggered it and serves as a transient lymphoid structure to recruit naïve lymphocytes from the blood and to promote their interaction with local antigens in the airways to favour activation and differentiation into effector cells. The iBALT is formed near the basal side of the bronchial epithelium and in close association with the pulmonary blood vessels in response to infection or other inflammatory stimuli [203]. iBALT formation is protective against acute viral and bacterial respiratory infections and it has been also shown to contribute during chronic *M. tuberculosis* infection. However, activation of lymphocytes in TLS can also contribute to airway damage. For instance, these structures tend to form near small airways in COPD patients and are associated with more severe forms of the disease [204].

Lung resident memory T cells (T_RM_) protect against infection by expanding rapidly upon challenge. CD4^+^ T_RM_ reside in the lung parenchyma, whereas CD8^+^ T_RM_ are found in parenchyma and the airway epithelium. CD4^+^ T_RM_ are maintained over longer periods of time while CD8^+^ T_RM_ decay and must be replenished from circulating T effector memory cells [205]. A subset of memory CD69^+^ CD103^+^ T regulatory (Tregs) cells expressing FoxP3 are also found in the lungs of mice and humans [206, 207]. Antigen-specific memory Tregs differentiate in response to certain respiratory infections such as influenza and can persist in the lungs after the pathogen has been cleared. These cells can contribute to limit the damage upon reencounter with the pathogen [207] and can limit the pro-fibrotic potential of CD44^hi^ CD69^+^ CD103^lo^ CD4^+^ T cells [208].

#### Metals and adaptive immune cells

Trace metals including iron and zinc, as well selenium can influence adaptive lymphocyte biology and function. As for most immune cells, iron is key to T and B lymphocytes. Both express TFR1 or CD71 which allows them to acquire transferrin-Fe^3+^ via endocytosis. The acquired iron is used as a cofactor in several enzymes and a small proportion is stored in ferritin or remains as part of the labile iron pool in the cell; although the iron reserve pool in lymphocytes is very limited compared to other immune cells [209]. In addition to TFR1, lymphocytes also express LfRs upon activation which are particularly abundant on ɣδ T cells [195].

Both iron overload and iron deficiency impact adaptive immunity and lymphocyte function but the different T helper and cytotoxic lymphocyte subsets display different responses to iron perturbations and their dependence on TFR1 iron uptake. Mice fed an iron-rich diet show lower IFN-ɣ production and impaired delayed-type hypersensitivity responses, whereas iron-deficient diets are linked to impaired T-cell proliferation [209]. In vitro, blocking TFR1 with antibodies causes the arrest of helper T cell (Th)1 cell proliferation, whereas Th2 cells do not seem to be affected. In contrast, supplementation of culture media with iron and transferrin boosts lymphocyte proliferation induced by polyclonal activators such as concanavalin A, phytohemagglutinin or LPS [210]. Supporting the importance of transferrin and iron for lymphocytes in the lungs, transferrin levels in the BALF of patients with COPD and sarcoidosis correlate with the number of lymphocytes in the BALF [211].

Iron levels are key to adaptive responses to pathogens and vaccines; anemia, iron or increased hepcidin and low serum iron caused by a mutation in TMPRSS6 predict reduced responses to rubella, diphtheria, pertussis, *H. influenzae* type B and pneumococcal vaccines [212, 213]. Importantly, in humans another homozygous mutation in *TFRC* (the gene encoding TFR1) results in defective TFR1 iron internalization. Homozygous carriers of this mutation display a combined immunodeficiency syndrome that results from impaired T and B cell proliferation, defective class-switching and lower antibody production, demonstrating the importance of iron uptake and TFR1 for adaptive response and lymphocyte’s function [214].

T cells are also affected by iron overload as seen in certain cohorts of patients with HH or transfusion-dependent thalassemia. A subgroup of thalassemia patients present with unusually low counts of CD8^+^ T cells, which can improve after subcutaneous chelation therapy with the iron chelator deferoxamine (DFO) [215]. A similar phenotype has been observed in HH patients. HFE competes with transferrin for binding to TFR1 and downregulating iron cellular uptake. Iron‐sensing via the HFE-TFR1 axis ultimately induces hepcidin transcription blocking iron efflux. HH is associated to the partial or total loss of hepcidin, which results in excessive iron uptake and accumulation in the tissues. HH patients present anomalies in CD8^+^ T cells and altered CD4^+^/CD8^+^ T cell ratios; in particular HH is associated with lower numbers of CD8^+^ T cells in the circulation and liver and a defect in the generation of CD8^+^ memory cells [197]. Altered cytokine profiles are also seen in HH patients, where CD8^+^ T cells increase the production of IL‐10 and IL‐4 contributing to Th2 polarization of the adaptive response. Although HFE is a non-classical MHC Ib molecule it does not appear to have antigen presenting capabilities; however, a role for HFE in the antigen presentation process cannot be ruled out. TFR1 interacts with the TCR ζ chain, which is involved in signal transduction upon antigen recognition [216]. Therefore, by competing with transferrin, and depending on iron availability HFE could regulate T cell activation. In addition to the altered lymphocyte ratios, some HH patients may have altered iNKT numbers. Iron overload was associated with reduced numbers of iNKT cells, which was more notable in untreated patients [200].

The balance between iron and selenium also seems important to the development of T cell responses. Iron metabolism is key to survival of CD8^+^ T cells as Fe^2+^ mediates a type of cell death known as ferroptosis in CD8^+^ T cells. During ferroptosis, Fe^2+^ reacts with H_2_O_2_ produced by mitochondrial respiration driving the formation of free hydroxyl radical (OH·) which promotes lipid peroxidation and death. The glutathione peroxidase 4 (Gpx4) is a selenoenzyme that acts as a major scavenger of phospholipid hydroperoxides and is essential to prevent ferroptosis. Gpx4 is essential for homeostasis of both CD4^+^ and CD8^+^ T cells, as mice lacking Gpx4 have impaired proliferate T cell responses during a viral challenge or *Leishmania* infection [217]. Selenium has been shown to be important for both cellular and humoral immune responses, although cell-mediated immunity seems to depend more on selenium. Selenium can enhance responsiveness of lymphocytes to IL-2 by inducing the upregulation of IL-2 receptors, therefore promoting proliferation, cytotoxic activity and antibody production [218]. High selenium diets promote Th1 differentiation in mice. Whereas selenium deficiency has been linked to more severe influenza in mice [219]. Selenium deficiency leads to reduction in the expression of selenoprotein K (SelK), which is expressed in the endoplasmic reticulum membrane of many immune cells including T cells. SelK deficiency impairs proliferation of T cells as it impairs crucial Ca^2+^ fluxes upon TCR activation. SelK deficiency also affects neutrophil and macrophage function increasing susceptibility to a viral challenge. Zinc (Zn^2+^) is required for thymic development of T lymphocytes. Zn^2+^ deficiency leads to impaired Th1 responses while promoting Th17 differentiation and IL-1β production [219].

## Host nutrient availability and the microbiome of the lung

The human body harbours over 100 trillion microorganisms that live in a commensal relationship with their host, mostly at barrier surfaces and mucosae including the gut, the airways, skin and genitourinary tract. Imbalances in the composition of the human microbiota have been increasingly linked to pathologies such as allergy, asthma, chronic inflammation and autoimmunity. While the gut has been the most studied and characterized niche in terms of the microbiome, in recent years the importance of other niches including the lung microbiome, became apparent. We are only now learning that the lung is colonized by a complex and dynamic microbiota. While progress in understanding how changes in the lung microbiome contribute to disease has been limited due to sampling, technical and analytical problems, 16S sequencing studies have demonstrated distinctive differences in the lung microbiome between health and disease [220]. Despite the low density of the lung microbiota, the extraordinary diversity of interacting microbiota is evident, and it is the change or decline in this diversity that is often associated with the progression of disease. To date, no particular bacterial genera have been implicated in lung disease and the variability in the diversity of the species detected in the upper versus lower respiratory tract as well as the regional variations in the host environment (e.g., mucus or surfactant secretion, pH, nutrient or oxygen availability) has also limited our understanding of the contribution of the respiratory microbiome in lung disease [221].

It appears that the airway microbiota in healthy lungs is dominated by *Bacteroidetes* and with prominent genera including *Prevotella*, *Veillonella* and *Streptococcus* [222–224], all of which are dependent on metals for growth and survival [9, 225, 226]. Approximately 30% of all proteins in bacteria depend on metals for their function [226] and changes in host metal availability alters bacterial diversity and abundance in vivo [227–236], however there have been few studies examining the effect of metal availability directly on lung microbe populations [237]. In addition, whether or not changes in host metal availability in the lung directly modifies the lung microbiome which in turn contributes to the development and progression of chronic or acute respiratory disease is an evolving question.

Similarly, it has become evident that it is not only the microbial communities but also the metabolites they produce that can influence host susceptibility to certain diseases [238]. Despite the advances in microbiome research, our knowledge of the airway microbiome is still way behind compared to that of the gut microbiome. And as such, lung microbiota composition, microbiota derived metabolites and how they could contribute to lung inflammation, immunity and disease is only in its infancy [239, 240]. Alterations in bacterial burden, gut microbial species and the metabolites they produce are associated with altered inflammation and immunity in the lungs as well as the development of lung diseases. This interaction, known as the gut-lung axis allows the access of gut-derived bacterial components, hormones, microbial metabolites, endotoxins and cytokines to the lung niche via the bloodstream, also influencing immune cell trafficking. Future research of the lung microbiota in the context of both heathy and diseased airways will likely discover the causes and consequences of altered lung microbiota in lung disease and identify microbial derived metabolites that play important roles in these processes. Whether or not microbe derived metal regulators interfere with the host immune responses in the lung therefore requires significant attention. In the below sections we will discuss the existing evidence for alterations in host metal availability in chronic respiratory disease and the evidence, if any, for the role trace metals have to play in the nutritional immune response or to changes in the lung microbiome.

## Nutritional immunity and the host lung microbiome in chronic respiratory disease

### Asthma

Asthma is a chronic and heterogeneous disease of the airways characterized by airway hyperreactivity, difficulty breathing, cough, wheezing and chest tightness. It is estimated that 300 million people are affected with asthma and that ~ 250,000 die as a result each year. The pathogenesis of asthma is complex with innate and adaptive cells acting together with epithelial cells to induce airway hyperreactivity. A broad clinical spectrum of phenotypes associated to different underlying immune mechanisms (endotypes) have been described [241, 242]. Asthma can be divided in two subsets: the eosinophilic type mainly controlled by Th2 or ILC2s, and the neutrophilic type which is characterized by a strong Th17 component and can present as steroid-resistant asthma. *Eosinophilic asthma* can be categorized as Th2^hi^ and Th2^lo^ component depending on the presence of IL-4, IL-5 and IL-13 being produced and the number of eosinophils in blood and lungs [243]. In addition to Th2 cells, ILC2 can also contribute to the pathology of asthma and produce high amounts of type 2 cytokines. These cells have been identified in samples of blood but also BALF and sputum of asthmatic patients [177]. ILC2 also drive mucus hypersecretion, mucus cell metaplasia, fibrosis and inflammation which are driven by IL-13, eosinophils and monocytes [244]. Late onset and steroid-resistant asthma phenotypes are associated with IL-17 driven neutrophilic asthma and present with a more irreversible airway obstruction. Besides Th17 cells, cell including ɣδ T cells, NKT cells and ILC3 can contribute to IL-17A, IL-17F and IL-22 production amplifying the pathology [242, 245].

T helper responses in asthma are supported by DCs that are directly activated by allergens and other environmental insults or via the damaged epithelium. Allergens including house dust mites, spores, cat dander and others can have protease activity that damages epithelial cells and triggers protease-activated receptors and triggers the release of damage associated molecular patterns (DAMPs) that lead to the secretion of innate responses via IL-33, TSLP, TLR4 and C-type lectin receptors among others [242]. Among lung DCs conventional DC expressing CD11b and SIRP1α and that rely on interferon regulatory factor (IRF)4 are the most important for allergic sensitization [246, 247]. DCs and epithelial cells not only contribute to sensitization to allergens but also play a role in ongoing asthma.

#### The lung microbiome and asthma

The time from the perinatal period up to the first years of life is key to the establishment of a “healthy” microbiome. The composition of the microbiome at all anatomical sites is dynamically shaped by the interactions between symbionts, pathogens, the immune system, nutrition and the environment. Disturbances during the critical period of establishment of this ecosystem may determine future pathological manifestations [248]. Several retrospective and longitudinal studies suggest this is the case for asthma and airway hyperreactivity, where aberrant immune development results from environmental exposures that may influence the airway microbiota. The analysis of the airway microbiome in infants revealed an association between the airway microbial composition and the risk of developing asthma within the first 6 years of life. Enrichment of the upper airway microbiome with taxa including *Veillonella*, *Prevotella*, and *Gemella*, was associated with increased risk of asthma and a characteristic immune profile in the airway which was also independently associated with increased asthma risk within 6 years. While the study cannot prove causation, it suggests a potential link between an early shift in the airway microbiome composition and immune perturbation which could lead to asthma later in life [249].

Analysis of different respiratory samples in healthy vs asthmatic adults suggests that the lungs of asthmatic patients receiving corticosteroid therapy are dominated by the phylum Proteobacteria which include the genera of potential pathogens including *Haemophilus*, *Moraxella*, and *Neisseria* [250]. Response to steroids in accompanied by an increase in Actinobateria, whereas *Kelbsiella* is linked to severe asthma [250]. In addition, patients with severe neutrophilic asthma receiving high doses of inhaled corticosteroids display lower airway microbiome diversity, enrichment with *Moraxella* and *Haemophilus* and a reduction in *Streptococcus*, *Gemella* and *Porphyromonas* [248]. While airway microbiome profiles seem to correlate with severity of the disease and may even predict response to corticosteroids, it is not possible to discriminate whether these changes are promoted by the interactions between commensals and the immune system or induced by the steroid treatment. In a group of steroid-naïve asthmatic patients, members of the Sphingomonodaceae family and species of *Haemophilus*, *Neisseria*, *Fusobacterium*, and *Porphyromonas* were enriched in the bronchi compared to healthy controls, whereas members of the *Mogibacteriaceae* family and *Lactobacillales* order were lower. Importantly, steroid treatment caused a shift in the balance of the bronchial microbiome and differences were linked to responsiveness to treatment [251].

In addition to the local airway and lung microbiome, the distant gut microbiome and the gut-lung axis of immune regulation have been implicated in the pathogenesis of asthma. Significant reduction in species of the genera *Lachnospira*, *Veillonella*, *Faecalibacterium* and *Rothia* in the gut of 3-month-old infants correlated with elevated risk of developing asthma [252]. Mechanistically, high risk of asthma and gut dysbiosis has been linked to loss of short-chain fatty acid (SCFA)-producing bacteria which ferment soluble fibre into these highly volatile metabolites (e.g., acetate, propionate, butyrate) known for their immunoregulatory properties [253] Higher levels of butyrate and propionate in the stool of 1-year old toddlers associate with less atopic sensitization and low risk of asthma between the age of 5 and 6 [254] and supplementation with soluble fibre inulin, associates with clinical improvement of asthma in adults and an increase in *Bifidobacteria* which degrade inulin into the SCFA acetate and lactate [255].

Intestinal bacteria can also produce metabolites that may be detrimental for lung function. For example, histamine-producing bacteria are more abundant in the gut of asthmatic patients compared to healthy controls and this histamine has been liked to airway hyperrreactivity [248]. Shifts in the microbial populations in the airways and in the gut seem to be associated with asthma endotypes and severity, the causal link between development of the disease and dysbiosis remains to be proven. The current evidence supports mechanisms of immune modulation mediated by microbial-derived metabolites. While an inverse correlation between SCFA in the gut and asthma severity exists, whether significant concentrations of SCFA can reach the bloodstream or the lungs remains questioned. It would be interesting to determine if changes in the lung microbiome influence local production of these metabolites to directly modulate the lung response to allergens and other environmental insults that could promote asthma.

#### Trace metals, nutritional immunity and asthma

Epidemiological studies have shown a correlation between iron deficiency and atopic disease, involving iron metabolism in the regulation of the immune response to allergens and atopic airway hyperreactivity [256, 257]. A large cross-sectional study in children and adolescents in the US, found a strong association between anaemia and atopic disease, including eczema or asthma [257]. The prevalence of asthma decreases with age and the iron reserve is higher in adults compared to children supporting the idea that the higher incidence of asthma and atopic disease in early life is linked to iron deficiency. In adulthood, men have higher levels of iron compared to women and asthma primarily affects women, again supporting a role of iron in prevention of asthma [41]. Though, the relationship between iron and asthma is not straightforward: increased iron stores in the form of ferritin have been associated with decreased odds of asthma, whereas higher tissue iron (lower serum soluble transferrin receptor) and lower body iron have been linked to lower lung function [256].

The iron storage capacity of Th2 cells is higher than Th1 as the latter have a lower iron labile pool [258]. Iron chelation affects Th1 cytokine production including IFN-ɣ, IL-12 and IL-18, but not Th2 cytokines [259]. Therefore, a limited iron supply during stimulation of lymphocytes by allergens may favour development of Th2 responses over Th1 and predispose to allergic sensitization [41]. Severe and moderate asthma patients present low cell-free (non-heme) iron levels in BALF which correlate with lower lung function measured as (forced expiratory volume in 1 s (FEV1), whereas iron-loaded cells numbers are increased and also show increased expression of *DMT1* and *TFR1*. These features were recapitulated by a murine model of house dust mite asthma where macrophages display the highest expression of *Tfr1* and also upregulate *Il13*, suggesting that by accumulating iron they orchestrate the type 2 response in the lungs [260]. The relationship between iron metabolism and pathophysiology of asthma represents a dichotomy: while low systemic and local iron levels in BALF correlate with severity of asthma, cellular iron overload is also linked to the pathology of the disease.

Importantly, most allergens of mammalian origin including those in dander, urine, fur, and saliva of animals, belong to the lipocalin family of proteins and have the capacity to sequester bacterial siderophore-iron complexes. Some of these allergens promote Th2 responses when not bound to iron, suggesting that iron deficiency may potentiate the stimulatory potential of lipocalin-like allergens [261, 262]. In addition, to promoting Th2 responses directly, allergens may change the microbial ecosystem of the gut and/or the airways by sequestering bacterial siderophores and altering the availability of iron to certain bacteria, therefore inducing dysbiosis. Supporting this notion, supplementation of dietary iron in rats favoured the production of the SCFA butyrate compared to animals fed an iron deficient diet [263]. This suggests that iron availability can influence the composition of the microbiome and the production of immunomodulatory metabolites such as SCFA that are important modulators of type 2 allergic responses and asthma [41].

The role of other metals in the pathophysiology of asthma is less clear but serum concentrations of trace elements including copper, zinc and selenium have been found to be altered in individuals suffering from asthma compared to healthy controls. Imbalances in selenium have been implicated in allergic inflammation. Curiously, while mice fed on high and low selenium diets show low incidence of allergic inflammation in an ovalbumin sensitization model, intermediate doses of selenium were associated with heightened inflammation. This suggests that the modulation of the allergic response by selenium in the lungs is not linear and may involve several intermediate enzymes and other unknown mechanisms [264].

Two independent studies reported elevated serum levels of copper in adults suffering from asthma compared to healthy controls, whereas zinc and selenium were lower and magnesium remained unchanged [155, 265]. While the mechanisms by which alterations in trace metal concentration contribute to the pathology are unclear, given the role of copper and zinc in regulation of redox metabolism, altered levels of these metals may contribute to imbalances in oxidative stress in asthma. Indeed, asthmatic patients present higher levels of NO and nitrated products than healthy individuals, and these correlate with the severity of the disease. Non-enzymatic decomposition of S-nitrosothiols (RNSO) is mainly catalyzed by Cu^2+^ ions leading to generation of NO and the corresponding disulphide [266]. Therefore, it is possible to postulate that elevated copper levels in asthma may accelerate RSNO consumption with the concomitant increase in NO. Supporting this hypothesis, RSNO deficiency in the airways has been linked to asthmatic respiratory failure in children which suggests that RSNO metabolism and in particular their copper-mediated catalytic decomposition could be therapeutic targets in asthma [267].

#### Chronic obstructive pulmonary disease

COPD is a chronic inflammatory lung disease associated with cigarette smoke or other environmental exposures. As a leading cause of death worldwide, COPD encompasses chronic bronchitis and emphysema [268, 269] and involves an aberrant immune and inflammatory responses to the inhalation of noxious particles [269, 270], with chronic inflammation persisting for many years after [204]. The definitive molecular mechanisms underlying the chronic inflammatory changes observed in COPD remain to be determined, however there is overwhelming evidence implicating AMs in this process [204, 271, 272]. AMs exposed to cigarette smoke have been shown to ineffectively clear respiratory pathogens, damaged epithelial cells as well as having defective responses to activating stimuli [273–276]. Strikingly, there is a 25-fold increase in the number of AMs in the lung parenchyma and alveolar space in COPD, which correlates with disease severity [277, 278] and areas of lung destruction [272], highlighting their central role in disease pathogenesis. Neutrophilic inflammation is also a hallmark of COPD and is associated with bacterial infection [279] whereas eosinophilic inflammation is present in a subgroup of COPD patients [280], and is associated with less bacterial infection [281]. Aberrant regulation of the immune response is a clear factor in the development and progression of COPD however little is known regarding the microbiome or nutritional immunity in this process.

#### The lung microbiome and COPD

Microbiome changes have emerged as a contributing factor in COPD progression, clinical phenotypes, severity and long-term mortality [282–284]. Studies comparing sputum and BALF microbiota between stable COPD patients and healthy controls have identified a change in microbial diversity with an increased abundance of *Moraxella*, *Streptococcus*, *Proteobacteria, Veillonella*, *Eubacterium* and *Prevotella* in disease [283–289]. Other studies of BALF report no difference in *Streptococcus* [290]. Conversely, increased *Proteobacteria* and reduced *Firmicutes*, *Bacteroidete*s, *Streptococcus*, *Haemophilus influenza* and *Prevotella spp*. have been documented in COPD lung tissue explants [291]. During an exacerbation event (periods of symptom worsening and reduced lung function) shifts in bacterial composition, characterized by a relative increase in *Proteobacteria* that falls in response to antibiotics have also been observed [292, 293]. Some of the above studies suggest that these changes may be independent of smoking status and that real changes in microbial diversity may be more apparent upon examination of associations in specific COPD endotypes [289] such as the neutrophilic inflammatory endotype [283, 294]. Intriguingly the faecal microbiome and COPD patients is also distinct from those of healthy individuals, with *Streptococcus sp000187445*, *Streptococcus vestibularis* and multiple members of the family *Lachnospiraceae* correlating with reduced lung function [295]. During an acute exacerbation event the faecal microbiome has also been shown to display a lower relative abundance of *Firmicutes* and *Actinobacteria* with an increase in *Bacteroidetes* and *Proteobacteria* [296]. The importance of the gut microbiota in COPD development is further highlighted by the reversal of murine smoke-induced inflammatory emphysema via faecal microbiota transplantation or a high-fibre diet, possibly via the beneficial effects of SCFAs [297].

#### Trace metals, nutritional immunity and COPD

Genome wide association studies of COPD patients implicate a role for abnormal iron metabolism in COPD [298–302]. Current and former smokers have abnormally high levels of iron in sputum, BALF in exhaled breath condensate, and in AMs, compared to non-smoking controls [211, 303–312]. Anaemia and non-anaemic iron deficiency often accompany COPD [313] with anaemia being an independent predictor of mortality [314–316], and with iron-deficient COPD patients having more exacerbations than control subjects [317]. Smoke exposure also reduces hepcidin expression in murine models. COPD patients have an inappropriate suppression of hepcidin in response to iron deficiency, with less hepcidin expression in severe end-stage disease [54, 316, 318]. This may or may not be related to findings that iron accumulates inside cells and tissues of in vitro and in vivo COPD models [304, 305, 309]. Whether increased cellular iron is pathogenic [319] or a protective [307, 320] stratagem should consider the lung and systemic iron regulation as separate entities. Both the use of iron chelators directly in the lung as well as administering intravenous ferric carboxymaltose to iron deficient COPD patients may have beneficial effects [319, 321].

As previously mentioned, iron plays an important role in the functional response of macrophages and other immune cells of the lung and similarly iron regulatory pathways play an important role in the response of these cells to smoke [54, 304, 319]. Specifically, cigarette smoke increases the expression of FPN, ferritin and the TFR1 on AMs and inhibits hepcidin induction by LPS as well as inducing ferroptosis [54, 322]. However, whether or not the above changes in macrophage iron regulation in the lung as a result of smoke or COPD progression alters the interplay between immune cells and the lung microbiome remains to be determined.

Zinc levels have also been shown to play an important role in COPD. COPD patients have lower levels of zinc [77, 323] and smokers with low dietary zinc intake have an increased incidence of COPD [324]. The zinc transporter ZIP8 is also increased at mRNA and protein levels in the lungs of chronic smokers [325] and zinc deficiency potentiates the effects of smoke on the epithelial cell barrier function [326, 327]. Depletion of zinc in adult mice resulted in a significant increase in lung cadmium burden and permanent lung tissue loss following prolonged smoke exposure [328]. Zinc also regulates the immune response to smoke whereby airway inflammation is exaggerated in zinc deficient mice [329] and zinc supplementation reduces AM numbers in smoke-exposed mice [330]. Zinc protects against cadmium toxicity and loss of zinc in AMs leads to the toxic accumulation of cadmium in the AMs of smokers [331]. Functionally, loss of zinc inhibits efficient efferocytosis of apoptotic epithelial cells by AMs [77]. Whether or not a loss in zinc levels alters the microbiome of the lung or the response of immune cells such as AMs to infection remains to be determined.

Copper also plays a role in the development of COPD. Copper deficiency induces emphysema in animal models [332–334] and the EBC of stable COPD patients contains lower copper levels than of controls an observation that is positively related to FEV1 in individuals with COPD [335]. Intriguingly, individuals with Menkes disease, an X-linked recessive disorder of mutations in the intracellular copper-transporter ATP7A display an increased incidence in emphysema [336, 337]. A loss of copper directly impacts AEC integrity and copper directly regulates elastin synthesis via the copper dependent enzyme lysyl oxidase [334, 338]. However, whether or not copper levels in lung immune cells dictate function or regulate responses to cigarette smoke remains to be determined. Finally, selenium responsive genes are altered in individuals with COPD [339] and COPD patients with higher selenium have a higher FEV1 [340]. Selenium and manganese levels may be beneficial before the clinical onset of COPD in smokers [341] and manganese levels are higher in COPD patients with severe disease when compared to control smokers [342] however the role of selenium or manganese in disease pathogenesis is unknown.

#### Cystic fibrosis

Cystic Fibrosis (CF) is an autosomal recessive disease caused by mutations in the CF transmembrane conductance regulator gene (CFTR). The *CFTR* gene encodes a protein that mainly acts as a chloride channel that transports ions across the apical membrane of epithelial cells. This disease is characterized by progressive lung disease, malnutrition, growth defects and several other presentations [343]. In the lungs, CF is characterised by mucus accumulation and obstructive lung disease. Reduced mucus clearance leads to higher bacterial loads in the lower airways and recurrent respiratory infections are the main cause of morbidity and mortality in CF patients, with *Pseudomonas aeruginosa* being one of most common pathogens in CF.

#### The lung microbiome and CF

The altered landscape of the CF airways results in a dysbiosis of the airway microbiome, however which specific factors of the CF lung microenvironment drive this change in the microbiota still remains unknown [344]. The onset of the CF microbiome involves an increase in bacterial burden and a reduction in microbial species diversity. Infants and children with CF have a higher microbial diversity which is lost with age, disease progression, and the domination of CF pathogens, most notably *P. aeruginosa* [345–349]. In advanced disease the lower airways consist of mostly homogenous populations of CF pathogens [350]. The main taxa found within CF microbiomes are *Streptococcus*, *Prevotella*, *Veillonella*, *Rothia*, *Actinomyces*, *Gemella*, *Granulicatella*, *Fusobacterium*, *Neisseria*, *Atopobium* and *Porphyromonas* [351, 352]. Diversity appears to serve as a marker for lung function as reduced species richness correlates with reduced lung function [351, 353]. Several studies have examined the airway microbiome in CF patients during exacerbation [351, 354, 355] and have observed that overall microbial community structure remains the same during stable periods and exacerbations and that the extent of changes observed during exacerbation are dependent on the community composition and diversity at baseline. In one study the relative abundance of *Gemella* was increased in most of the patients during exacerbation and was the most altered genus between baseline and exacerbation [354]. In this same study a subset of patients had substantial changes in microbial community structure during exacerbation in that those that had *Pseudomonas* dominated microbiota communities at baseline became more diverse at exacerbation. Furthermore, the presence of anaerobes in the microbiota is associated with reduced inflammation and higher lung function at early exacerbation compared to *Pseudomonas* [356]. While many studies have identified the constituents of the microbial community in the CF lung and during exacerbation and stable periods, there are few mechanistic insights into how these microbial residents of the airways can contribute to this disease. A recent study quantified bacterial active translation in the sputum from CF patients and found that active bacteria (i.e., those actively translating proteins) represent only a subset of those captured by conventional sequencing [357]. By adopting a dormant phenotype in which reduced cellular activity endows a temporary multi-drug resistant phenotype, the inactive bacteria subpopulations could persist during antibiotic challenge. While the common residents of the CF airway microbiota are now known, their physiology and how they interact with other members of the microbiota and with their airway microenvironment remain to be more fully explored in order to understand how CF drives altered microbial community in the airways and how the CF microbiota subsequently contributes to CF disease progression.

#### Trace metals, nutritional immunity and CF

Systemic iron deficiency is prevalent among adults and children with CF [358–364]. However iron levels are elevated in the airways in CF with higher iron levels detected in the sputum and within AM and IMs [365–368]. With macrophages being key regulators of extracellular iron levels, they represent a likely source contributing to increased airway iron. Indeed, CF macrophages have recently been shown to have altered iron metabolism that can be corrected with CFTR modulators ivacaftor and lumacaftor [369]. Furthermore, airway epithelial cells expressing ΔF508-CFTR, the most common CFTR mutation observed in CF, have been shown to have altered iron homeostasis and release more iron than those expressing WT-CFTR [370, 371]. This increased iron in the airways provides easier access of this vital nutrient to bacteria present in the airways, including *P. aeruginosa*. Iron not only is essential for growth, but it also regulates biofilm formation in this respiratory pathogen [369, 370, 372]. This has a major impact on the ability of these bacteria to persist in the lungs as bacteria within biofilms are much more difficult to clear by host immunity and by antibiotic treatment. With persistent *P. aeruginosa* lung infections driving increased mortality in CF patients, reducing high iron levels in the lungs or depriving *P. aeruginosa* of iron appears to be a sensible strategy to control this CF pathogen. Gallium has been used to treat *P. aeruginosa* in CF, this metal was shown to and inhibit growth of *P. aeruginosa* in sputum and improved lung function in people with CF and chronic infection by interfering with bacterial iron metabolism [373]. Gallium compounds have also been shown to inhibit in vitro and in vivo growth of *Mycobacterium abscessus* by disrupting iron uptake in this emerging important pathogen in CF[374].

Zinc deficiency has been observed in CF patients, especially those with malabsorption and impaired growth. The serum or plasma levels of subsets of CF patients, both child and adult, have been observed to be lower than healthy controls in several studies [375, 376]. Zinc supplementation has been used to treat CF patients, but studies have shown conflicting results, some have shown that zinc supplementation leads to improved lung function and reduced need for antibiotics and hospitalisations, while others showed no benefits for zinc supplementation [377–381]. Interestingly, while zinc levels in serum can be low, zinc levels in the airways of CFs are reported to be higher [382, 383]. The prevalence and the impact of altered zinc homeostasis in CF is not yet well defined, however zinc levels affect microbial community structure in CF. Explants of CF lungs had *S.aureus* and *P. aeruginosa* co-existing in calprotectin enriched regions. As calprotectin chelates zinc, regions in the lungs enriched with this protein will have lower zinc levels which causes *P. aeruginosa* to reduce its production of antistaphyloccal factor thus allowing for the co-existence of this bacterial species [384].

Studies have found reduced activities of copper enzymes from cells (mononuclear cells, neutrophils and erythrocytes) in CF patients, possibly indicating reduced copper availability within these cells [385, 386]. Interestingly copper supplementation does not counteract this copper deficiency in CF patients indicating an altered copper metabolism that cannot be corrected simply by supplementation [387]. One study reported that copper levels are elevated in the sputum in CF [382]. The cause and consequences of altered copper homeostasis in CF are yet to be determined. Selenium deficiency is observed in CF patients [388–391] but the levels of selenium in the CF lung have not been reported.

#### Non cystic fibrosis (CF) Bronchiectasis

Bronchiectasis refers to the permanent widening of the bronchi and usually presents clinically with coughing, sputum production and recurring respiratory infections, along with other symptoms. The widening of the bronchi leads to impaired mucociliary clearance and failure to effectively clear microbes and mucus leading to persistent infection and inflammation. Bronchiectasis is complex and heterogenous and can be the final common feature of many infectious, inflammatory, and allergic disorders [392].

#### The microbiome and bronchiectasis

Several studies of the lung microbiome in bronchiectasis have observed alterations. A longitudinal study of non-CF bronchiectasis patients found that the airway microbiomes remained stable over time and that patients with lower diversity of the microbial community were more likely to experience a subsequent decline in lung function [393]. This study also reported that the microbiomes of patients that were dominated by *Pseudomonas* differed greatly from patients whose microbiomes are *Haemophilus* dominated and that antibiotic treatment did not affect their microbiome composition. However, a larger study observed that disease severity is reduced with lower microbiome diversity [394]. Another study reported no significant changes in microbial diversity and during or after exacerbations, but also observed stability in the microbiomes of patients over time (6 months) even with antibiotic therapy [395]. This study also highlighted the differences in culture versus sequencing to identify the composition of the airway microbiota, notably *H. influenzae* is not detected well by culture. A study which compared bronchiectasis patients treated with low dose of a macrolide antibiotic (erythromycin) with a placebo control found that patients with *Haemophilus* dominated microbiomes were associated with fewer exacerbations but both *Pseudomonas* and *Haemophilus* dominated microbiomes were associated with lower lung function [396, 397]. Infections caused by non-tuberculous mycobacteria (NTM) are often seen in patients with bronchiectasis. These bacteria are found in the environment and usually do not cause disease but can establish chronic infections in people with underlying conditions. The role of NTM in the lung microbiome and how it may interact with other species in the microbiota of bronchiectasis patients, or even in healthy individuals, is not yet known. Limitations of sequencing approaches commonly used contribute to this lack of knowledge and studies have shown that 16S rRNA gene sequencing is not sensitive enough for *Mycobacteria* [398, 399]. Using a more sensitive technique for mycobacteria, a study was able to identify a non-tuberculous ‘mycobacteriome’ in in the mouth and upper respiratory tract of healthy individuals [399]. A study involving non-CF bronchiectasis patients with high prevalence of NTM used this mycobacteriome sequencing approach for the lower airways of 20 patients [398]. They found that in the lower airways of NTM positive patients, taxa identified as oral commensals were associated with increased inflammatory biomarkers. Studies with larger cohorts and using sensitive sequencing methods for mycobacteria are required to further investigate the role of NTM in the microbiome, particularly in the context of bronchiectasis.

#### Trace metals, nutritional immunity and bronchiectasis

The roles that metals such as iron, zinc and copper may play in non-cystic fibrosis bronchiectasis is not known and there are only a few reports measuring the levels of these metals in patients with this chronic lung disease. Some reports have observed that altered trace metals levels in non-CF bronchiectasis patients. Specifically, serum zinc levels have been reported to be lower in bronchiectasis patients when compared to healthy controls, but an earlier study did not find significant differences in serum zinc levels and found that even though zinc supplementation resulted in increased zinc serum levels no clinical improvement was observed [400, 401]. Two studies have detected higher levels of iron and zinc in the sputum of non-CF bronchiectasis patients compared to controls [382, 383]. Bacteria that cause recurrent infections in non-CF bronchiectasis could benefit from easier access to essential trace metals in the airways, supporting their growth. Thus further study of the source of these trace metals in the airways and their role in both the pathology of non-CF bronchiectasis, and in respiratory infections in those with chronic lung disease needs to be further investigated.

### Lung cancer

Lung cancer is one of the most aggressive and lethal cancers, with low treatment success and many complications. The most common form of lung cancer is non-small cell lung cancer which is subdivided into three main types; adenocarcinoma (AD) which arises in the peripheral bronchi, squamous cell (SC) which arises in the main bronchi and large-cell undifferentiated carcinoma [402]. The less common form of lung cancer is small cell lung cancer (SCLC) which accounts for ~ 15% of lung cancers is characterised by its neuroendocrine features [403]. Previously, lung cancer was thought to be non-immunogenic due to failed immunotherapies but has recently been revisited as being correlated with various immune cells and responses, particularly with inflammatory responses being integral to cancer progression [404]. Commonly during early stages of lung tumour generation an influx of immune cells such as leukocytes into the tumour and around the tumour microenvironment (TME) are observed [405]. The main source of this immune inflammation comes from M2 macrophages, which have been shown to promote angiogenesis, tissue remodelling and repair for the malignant cells [406].

#### The microbiome and lung cancer

Studies testing patients with lung cancer found strong associations between the presence of malignancy and high densities of *Haemophilus influenzae, Acidovorax, Klebsiella, Moraxella catarrhalis, Mycobacterium tuberculosis* and *Granulicatella adiacens *[224, 407]*.* Furthermore, comparison between the taxa present in SCLC and AD revealed specific taxa such as *Acidovorax*, *Klebsiella*, *Rhodoferax*, *Tepidimonas* and *Anaerococcus* were more abundant in SCLC [408]. Possibly through an environmental influence, the density of commensal bacteria was reduced within the lungs, allowing for more insidious opportunistic species to proliferate and drive lung inflammation. Microbial profiles have been suggested as a potential biomarker for lung cancer. Using 16S RNA sequencing and real time PCR Yan et al. (2015) successfully linked microbiota present in the saliva to lung cancer. Both *Capnocytophaga* and *Veillonella* were significantly higher in patients with lung cancer [409]. However, the sample size of this study was relatively small so further investigations into the affiliation between microbial composition in saliva and lung cancer is needed. In more recent studies, comparisons of the microbial profile between patients with emphysema and or lung cancer noted that the microbial composition of lung cancer tissue samples were distinctly different from both controls and patients with emphysema [410]*.* Furthermore, preliminary analysis of the microbiome in the sputum identified six further bacterial species that were significantly more abundant in lung cancer samples compared to controls [411]. It is clear that there is link between microbial composition and disease status, however, it is noted that the above studies attempted to analyse the lung microbiome indirectly through analysing the microbial composition in either saliva or sputum. Further direct analysis of the lung microbiome are needed to establish a stronger link between the microbial profile within the lung and lung cancer.

The gut microbiome has also been identified to play potential roles in the pathobiology of lung cancer. For example, *H.pylori* has been suggested to play a role in lung cancer pathology [412, 413] with patients with lung cancer possibly having a significantly higher rate of seropositivity for antibodies against *H.pylori* [414], however these findings have not been well replicated [415, 416]. The mechanisms behind bacterial burden and lung cancer include; the production of bacterio-toxins and pro-inflammatory factors, which could ultimately lead to the DNA damage and mutation causing malignancy, as well as a hyperinflammatory immune response [417, 418]. Also of note, evidence from patients with lung cancer has suggested antibiotic use during treatment to have a largely negative effect on tumour regression, with antibiotics only strengthening the pathogenic bacteria and staving off healthy commensal species [419]. Taken together it is clear that both commensal and infiltrating bacteria play a strong role in the carcinogenesis of lung cancer. Further investigations should attempt to directly assess the lung microbiome, for example, through BALF samples collected via endotracheal tube extraction to avoid contaminants from both mouth and upper airways [349].

#### Trace metals, nutritional immunity and lung cancer

Disturbed metal homeostasis has been previously proposed as a biomarker of lung cancer [420]. Specifically, increased level of copper and decreased level of zinc in the serum has been a successful indicator for lung cancer when compared to healthy controls [421]. Increased serum copper may aid in tumour progression through angiogenesis, while decreased zinc may lead to unprecedented changes in cell cycles and apoptosis [422]. In contrast, increased plasma zinc levels correlated with a lower risk of lung cancer [423]. Higher levels of zinc were also shown to regulate eight different cancer genes and suggested to prevent cancer progression through decreasing telomere attrition [423].

Iron accumulation has been seen in several cancers and has even been suggested as a potential target for cancer therapy [424]. In the context of lung cancer, ferritin levels in patients are significantly elevated when compared to controls and patients with COPD [425]. Increased expression of other iron regulators like LCN2 and TFR1 have also been observed in lung cancer patients [426, 427]*.* Furthermore, down-regulation of LCN2 or TFR1 in murine models for adenocarcinoma was shown to significantly suppress the growth of the tumours [426, 427]. Higher IL-6 levels in lung cancer patients [428] may be associated with the upregulation of hepcidin, resulting in decreased iron influx and induced cancer-related anaemia [429]. miR-20a, upregulated in non-small cell lung cancer (NSCLC), and can negatively regulate FPN, which may promote proliferation of cancer cells indirectly through the retention of iron [430]. Iron has also been reported to induce cancer stem cells and aggressive phenotypes through ROS generation in human lung cancer cells [431].

The TME has been characterized as a key player in tumour growth and progression [432]*.* Tumour associated macrophages (TAMs) are the most commonly found immune cell in the TME and have been shown to integrate an M2 ‘like’ phenotype to promote tumour growth and proliferation [405]. In contrast, a subset of TAMs, iron-loaded TAMs (iTAMs), were characterized as tumoricidal due to their ability to switch the polarization of M2 macrophages to pro-inflammatory M1 macrophages when exposed to iron or heme [433, 434]*.* Furthermore, AD patients with higher levels of iTAMS were shown to have an overall better survival in comparison to controls [435]*.* Little is known about where the surplus of iron is coming from, although some studies have suggested cigarette smoking may be a contributing factor [304]*.* Metagenomic sequencing of microbial profiles in the sputum of lung cancer patients have also identified increases in iron siderophore sensors and receptors [411]. M2 ‘like’ TAMs produce higher levels of FPN and lower levels of ferritin when compared to M1 macrophages. Furthermore, it was suggested that M2 ‘like’ TAMs contribute to tumour progression through their “iron-recycling” phenotype and fulfil the high iron demand from tumour cells by exporting iron into the TME [433]. Interestingly, the treatment of cells with exogenous iron can prevent liver metastases by inducing a pro-inflammatory M1 polarization in macrophages [436].

Links between other trace-elements, such as selenium and manganese with cancer have also been explored in the literature. While there are no reports on altered levels of manganese in lung cancer, some studies have reported an increase in manganese SOD (MnSOD) activity to be correlated with the disease [437–439]. Genetic analysis demonstrated that polymorphisms in the MnSOD gene were shown to contribute to a higher risk of lung cancer [440]. It is thought that these polymorphisms accelerate tumour progression due to the reduced ability to defend against oxidative stress. In more recent studies, over expression of an isoform of MnSOD in cancer cells was shown to increase tumour invasion in vitro [441]. The altered expression and mutations of MnSOD seen in lung cancer implicate an important role for mitigating oxidative stress in order to supress tumour progression.

Finally, the role of selenium in the pathogenesis and progression of lung cancer is controversial with some studies demonstrating a lower risk of lung cancer with higher exposure to selenium [442] and others reporting no correlation between dietary selenium and lung cancer risk a [443, 444]. Further metallomic and observational analysis are needed to clarify whether these metals are associated with the pathogenesis of lung cancer.

### Idiopathic pulmonary fibrosis

Idiopathic pulmonary fibrosis (IPF) is a form of interstitial lung disease, characterised by a gradual, irreversible decline in lung function [445]. Symptoms include gradual dyspnea and a non-productive cough. The disease is diagnosed via CT or lung biopsy to show the presence of usual interstitial pneumonia. As ‘idiopathic’ would suggest, the exact cause of the disease is unknown. IPF tends to affect those aged 60 and above, often with a history of smoking, and is more common in men than women. Environmental, occupational and genetic risk factors may also play a role in the onset of IPF. It is thought that persistent damage and abnormal repair to the alveolar epithelium drives the disease. Treatments for IPF are limited, and none can reverse the fibrosis. The current strategy focuses on slowing the progression of the disease and two antifibrotics have been approved, nintedanib and pirfenidone [446].

#### The microbiome and IPF

Active infection in patients with IPF is a high risk for mortality, and as antibiotics can improve quality of life for IPF patients, this would suggest that the microbiome can negatively affect disease progression [447, 448]. One study showed bacterial burden in BALF from IPF patients to be twice that of healthy controls, and this increase has been shown to correlate with worsening disease progression [449, 450]. The microbiota of IPF patients has been shown to be less diverse than healthy controls, with increased number of *Streptococcus*, *Haemophilus*, *Neisseria*, and *Veillonella* [449]. The *Streptococcus* and *Staphylococcus* taxa in particular may be linked to increased mortality, though subsequent studies have not supported this [449, 451, 452]. Instead, it may be the increased bacterial load, rather than particular species that is detrimental [449]. Interestingly, germ free mice had reduced mortality in a bleomycin-induced pulmonary fibrosis model despite similar levels of fibrosis, with the authors of the study speculating that the microbiome may alter the balance between Th1 and Th2 responses [450]. In a study comparing the difference in the microbiomes of patients with either stable IPF or those undergoing an acute exacerbation, those with stable IPF had a reduced bacterial load in their BALF fluid [452]. With increased bacterial load comes epithelial cell damage and increased expression of pro-inflammatory cytokines leading to inflammation and fibrosis [453]. Genetics may play a role determining the increased bacterial burden. Mucin 5B is one of a family of proteins that when combined with water forms mucus. Individuals with a polymorphism in the promoter region of the *MUC5B* gene, a gene associated with susceptibility to IPF have a higher bacterial burden than those lacking this polymorphism [449].

As bacteria must acquire metals essential for their viability from their host the altered metal profile in the lung during IPF, as discussed below, may affect bacterial growth. *Streptococcus pneumoniae*, for example, requires manganese and iron from its host, while high levels of copper are toxic [454]. Growth of both *Haemophilus influenzae and H. parainfluenzae* can be inhibited by the addition of the iron chelator, deferoxamine, to the growth media [455]. Whether the changes seen in metal availability in the lung drive infection during IPF, or whether they occur due to infection or the disease itself is unknown. Further study would be required, but as metal availability can affect the growth of the bacterial species that may exacerbate IPF, monitoring metal levels in BALF may be an indication of how susceptible patients with IPF are to bacterial infection.

#### Trace metals, nutritional immunity and IPF

Exposure to different metals, whether in an environmental or occupation settings, has been linked to the development of lung fibrosis and is considered a risk factor in the development of IPF [456]. The availability and metabolism of several metals have also been implicated in IPF. BALF from IPF patients showed lower levels of chromium, nickel, manganese and zinc when compared to healthy controls, and the authors of this study speculated that this may due to increased oxidative stress as these metals are involved in the antioxidant response [457]. Of the metals implicated in IPF, iron has been the most widely studied. Iron homeostasis is known to be altered in IPF patients as increased iron deposition is seen, which may also lead to increased oxidative stress in the lung [458, 459]. The same study also showed iron and copper to be elevated in BALF of IPF patients [457].

AMs may play a role in driving fibrosis [460]. Iron accumulation in macrophages leads to persistent activation in other diseases, and as iron is known to accumulate in AMs this may also contribute to damage in IPF [461]. BALF cells from IPF patients have increased iron-dependent ROS generation [461]. A higher proportion of AMs in IPF patients lack expression of TFR1, when compared to healthy controls [462]. Consequently, a higher level of transferrin is found in BALF and TFR1^neg^ AMs were shown to be unable to take up transferrin. The inability to sequester iron may lead to an increase in bacterial growth in the lung, for example *Staphylococcus aureus* can use transferrin bound iron as a growth factor [463]. The impairment in phagocytosis seen in these TFR1^neg^ AMs may also allow for increased bacterial growth. Coupled with this possible increase in bacterial burden due to a lack of TFR1 expression, the TFR1^neg^ AMs also had an altered gene expression profile that could be described as profibrotic. This increased proportion of TFR1^neg^ AMs was also linked to reduced survival in IPF patients [462].

Other metalloproteins may also contribute to fibrosis. Lysyl oxidase-like 2 (LOXL2), a copper-dependent monoamine oxidase, has been linked to pulmonary fibrosis [464]. LOXL2 plays an essential role in matrix remodelling and fibrogenesis, but in both bleomycin treated mice and patients with IPF, LOXL2 expression is increased [465, 466]. The transforming growth factor (TGF)-β/Smad signalling pathway has been implicated in the onset of IPF, and signalling through this pathway in fibroblasts from bleomycin challenged mice was reduced when LOXL2 was silenced with siRNA [464]. Targeting LOXL2 with an inhibitory monoclonal antibody has also been shown to be protective in bleomycin exposed mice [467]. SODs convert superoxide radicals into the less damaging hydrogen peroxide and oxygen [468]. Mammalian cells possess three isoforms: the cytosolic copper-zinc SOD1, the mitochondrial manganese-dependent SOD2, and the extracellular copper-zinc SOD3. Macrophages from patients who had been exposed to asbestos have high levels of the copper, zinc-SOD/SOD1, and produce high levels of H_2_O_2_, while SOD1^−/−^ mice were protected from developing pulmonary fibrosis following intratracheal exposure to asbestos [469]. Follow up studies showed that SOD1, via its generation of H_2_O_2_, drives a M2 macrophage phenotype among AMs, which is associated with the development of fibrosis [469, 470].

### Pulmonary arterial hypertension

Pulmonary arterial hypertension (PAH) is characterized by increased pulmonary arterial pressure associated with remodelling of the pulmonary arteries that leads to right ventricular hypertrophy and failure increasing the incidence of right heart failure and death [471]. Variants of pulmonary hypertension (PH) affect over one hundred million people globally [472]. Immune cells play an important role in the development of PAH supported by the observation that dysregulated lymphocytes, AMs, DCs, and mast cells frequently accumulate in the perivascular regions and in the pulmonary arterial vascular lesions found in human PAH tissue samples [473].

#### The microbiome and PAH

There have been few studies assessing the airway microbiome in individuals with PAH. However, a recent study described the difference in abundance of microbiota in pharyngeal swabs showing a significantly higher proportion of *Streptococcus*, *Lautropia*, and *Ralstonia* in patients with PH than reference subjects [474]. Gut microbiota differences have also been noted in PAH and experimental models for PAH have documented alterations in faecal microbiota composition with rodents displaying a three-fold increase in *Firmicutes*-to-*Bacteroidetes* ratio [475, 476].

#### Trace metals, nutritional immunity and PAH

Iron deficiency and/or anaemia is prevalent in PH patients, significantly affecting morbidity and mortality of PH patients [477, 478]. Similarly PH is a major cause of morbidity and mortality in patients with hemoglobinopathies and chronic haemolytic anaemias including sickle cell disease (SCD) and thalassemia [479]. Mutations (G208C) in NFU1, a mitochondrial protein that is involved in the biosynthesis of iron-sulfur clusters develop PAH in ∼ 70% of cases [480]. Iron accumulates in the lungs and AMs of individuals with PH [481]. Iron handling, in particular the hepcidin/FPN axis has been shown to be important in the vascular remodelling and endothelial cell dysfunction associated with PAH and loss of the iron regulatory protein Irp1 leads to the development of PH in mice [482–484]. While the role of iron in vascular endothelial and smooth muscle cells has been well studied; little is known regarding the role of iron in the perivascular immune cell infiltrates observed in PH. Circulating macrophages recruited to the lung that contribute to pulmonary vascular remodelling in PH have a distinct iron and HO-1 rich phenotype driven by haemoglobin [485], however whether other immune cells also display alterations in iron handling remains to be determined.

Increases in both zinc and copper transport have also been implicated in pulmonary vascular homeostasis [486, 487] and selenium levels may be lower in individuals with PH [488]. Pulmonary hypertension can be triggered by chronic hypoxia. Hypoxia has been shown to cause an NO-dependent increase in labile zinc in mouse lung endothelial cells from isolated perfused lungs (IPL) [489]. Hypoxic restriction is attenuated in IPL from mice treated with a zinc chelator suggesting a role for chelatable zinc in modulating hypoxic pulmonary vasoconstriction. Furthermore, zinc homeostasis has been implicated in PH as disruption of the zinc importer ZIP12 (Slc39a12) gene attenuates the development of PH in rats housed in a hypoxic environment [486]. Increased expression of copper-related genes, copper uptake transporter (CTR1), copper efflux pump ATP7A, and lysyl oxidase (a copper-dependent enzyme), was observed in mice with hypoxia induced PAH. Increased expression of CTR1 has also been observed in response to hypoxia within macrophages [490]. However, administration of a low copper diet in experimental models of PH had no effect on right ventricular (RV) failure [491]. Dietary administration of selenium to broiler chickens prevented RV hypertrophy associated with PH syndrome [492] suggesting some possible benefit for PH. Little is known regarding the role of these trace metals in immune cell function in the lung or interaction with the airway microbiome in PAH.

### Mycobacterial lung infections: tuberculosis and non-tuberculous mycobacteria (NTM)

Lung infections caused by mycobacteria cause a significant global health burden. *Mycobacterium tuberculosis (Mtb)*, the causative agent of tuberculosis (TB), causes 9 million new infections each year and over 1 million deaths [493]. While *Mtb* is a human adapted pathogen, non-tuberculous mycobacteria (NTM) consist of over 200 species of environmental mycobacteria, some of which can opportunistically infect humans. NTM lung infections most often occur in those with underlying conditions including COPD, cystic fibrosis and bronchiectasis. NTM infections are less prevalent than TB but the prevalence of these infections is increasing globally [494]. This is concerning as the treatment for NTM requires multi-drug regimen lasting over a year with only a 50–80% success rate [495]. Furthermore, recurring infections after treatment completion are common.

#### Mycobacteria and trace metal acquisition

Pathogenic mycobacteria must be able to acquire nutrients to support growth, survival and persistence within the host [496]. These intracellular pathogens acquire iron by stealing it from their host through the production of siderophores as well as utilisation of host iron-binding proteins heme, haemoglobin and transferrin [497]. Siderophores are compounds produced by some microorganisms with a high-affinity for iron in response to iron deficient conditions [498]. There are three types of mycobacterial siderophores: mycobactin, carboxymycobactin and exochelin. Mycobactin is associated with the bacterial cell envelope and transports iron through the cell envelope into the cytoplasm. The structure of mycobactin varies between bacterial species. However, its general lipid-soluble structure allows the binding of one iron atom per molecule at a very high affinity [498, 499]. Carboxymycobactin has a very similar structure to mycobactin but it is modified by the addition of a carboxylic acid group, making it more hydrophilic, thus it is an extracellular siderophore that is secreted by the bacteria. Exochelins are extracellular siderophores and are produced only by fast growing mycobacteria. Their structure consists of ornithine-derived hydroxamates as iron coordination sites allowing them to acquire iron from insoluble sources such as ferric oxide and ferritin [499].

Mycobacteria also have alternative mechanisms to siderophores to obtain iron from their host [500]. Heme is one of the largest human iron stores and as a result it is often exploited by bacteria as a source of iron. *M. tuberculosis* is capable of importing heme and either extracting the iron if needed or storing the heme. *M. tuberculosis* also requires heme itself and encodes its own heme biosynthesis pathway, which is essential if an external heme source is not provided [501–503]. Mycobacteria can also use haemoglobin as an iron source to support their growth. The growth supported by haemoglobin was accompanied by an decrease in siderophore production, suggesting an alternative iron-acquisition system [504].

Pathogenic bacteria must be able to safely store intracellular iron once it has been acquired from the host and like vertebrates, they use ferritins and bacterioferritins (Bfrs) to do so. Typically, ferritins assemble into spherical particles or ‘nanocages’ consisting of 24 subunits with a ferroxidase centre within each subunit. *M. tuberculosis* has both ferritin and Bfrs (BfrA and BfrB). The expression of *bfrA* and *bfrB* genes is regulated by the iron sensitive regulator IdeR such that ferritins levels adapt to the level of iron [505]. Deletion of BfrA and BfrB from *M. tuberculosis* results in a decreased ability to withstand oxidative stress, antibiotic treatment and survival within human macrophages. Loss of both genes leads to reduced virulence in a guinea pig model of TB infection and loss of *bfrB* lead to an inability for *M. tuberculosis* to persist in mice [506, 507]. These studies show the iron storage system of *M. tuberculosis* to be an attractive drug target, as it not only is required for full virulence but also to withstand antibiotic treatment (including the front-line TB drug isoniazid). *M. avium* encodes only one ferritin homologue (BfrA) which is less important to iron homeostasis and virulence in *M. tuberculosis*. As *M. avium* most often causes infections in those with chronic lung diseases such as COPD, CF and non-CF bronchiectasis where iron levels in the lungs are increased, one could speculate that iron storage within M*. avium* is particularly crucial to persist in the host and thus we could speculate that the BfrA homologue in *M. avium* could be important for this opportunistic pathogen in dealing with abundant iron. Interestingly, the crystal structure of Bfr from *M. smegmatis* (which is 87% identical to its homologue in *M. tuberculosis*) revealed that it contained zinc in its di-nuclear metal binding site [508]. The biological implications of this and whether this is also true for the Bfrs in other mycobacteria have not yet been explored.

#### TB and the microbiome

Studies have shown that *M. tuberculosis* infection affects both the lung and the gut microbiome of its host. However, the role of other mycobacterial species on the microbiome still needs to be elucidated. Interactions between the microbial communities of the gut and lung of an infected host can influence the outcome of *M. tuberculosis* disease progression and response to treatment [509]. The microbiota of sputum samples from TB patients were more diverse compared to respiratory secretions of healthy participants. Bacteria such as *Pseudomonas* and *Cupriavidus* were exclusive to TB patients and could influence the onset or development of infection [510]. BALF samples of TB patients showed a similar diversity in the lower respiratory tract with *Cupriavidus* bacteria possibly acting as a cofactor to secondary TB infections [511]. Furthermore, oral microbes present in the lungs of HIV patients on anti-retroviral therapy produce SCFAs, such as butyrate and propionate that increased patient susceptibility to *M. tuberculosis* infections by inhibiting the production of IFN-ɣ and IL-17A cytokines [512].

Mice infected with *M. tuberculosis* by aerosol lost their gut microbial diversity and recovered with a different microbial composition [513]. Furthermore, the administration of first-line TB antibiotics almost immediately (within 1 week) altered the intestinal microbiota of *M. tuberculosis* infected mice with distinct and long lasting effects [514]. Faecal samples from clinical pulmonary TB patients had dysbiosis of their gut microbiota with a significant decrease in the SCFA-producing bacteria. A decrease in SCFA-producing bacteria have also been found in systemic inflammatory disease and therefore, loss of these bacteria in TB patients may indicate systemic inflammation and an impaired immune response [515]. There have been attempts to create a classification model using the abundance of gut microbes to discriminate between healthy and diseased patients [515].

#### Trace metals, nutritional immunity and mycobacteria

Elemental analysis of the phagosomes of peritoneal macrophages of C57BL/6 mice infected with *M. avium*, *M. tuberculosis* or *M. smegmatis* showed that the iron concentration of the phagosomes containing the pathogenic mycobacteria was significantly higher [83]. Radioactive iron-loaded transferrin was used to reveal that the infected macrophages acquire extracellular iron and delivers it to vacuoles containing *M. avium* through the transferrin receptor. However, when the macrophages were activated with IFN-ɣ before infection, iron accumulation by the pathogenic mycobacterial phagosomes was prevented. IFN-ɣ is known to downregulate the transferrin receptor in order to reduce the iron pool and enhance macrophage activity. Furthermore, when the macrophages were treated after infection, there was no change in iron concentration indicating macrophage anergy to IFN-ɣ activation [83]. Iron is extracted from transferrin by mycobactins but holo-transferrin can also be taken up by *M. tuberculosis* within macrophages [516].

The *Nramp1/Slc11a1* gene is associated with the controlling intracellular pathogens such as *Mycobacteria*, *Salmonella* and *Leishmania*. Several studies have reported polymorphisms in *Nramp1* which are associated with increased susceptibility to tuberculosis and leprosy (caused by *M. leprae*) in humans [517–519]. NRAMP1 is a transmembrane protein localised to lysosomal membranes of phagocytic cells and is recruited to the phagosome upon infection [520–522]. It belongs to a family of divalent cation transporters and functional studies have shown that it transports iron out of the phagosome [523–526]. A recent report found that NRAMP1 restricts *Salmonella* growth in mice through magnesium deprivation, but whether this is via magnesium being directly transported by NRAMP1 has not yet been determined [527]. NRAMP1 is also known to have roles in nitric oxide production, phagosome maturation and induction of lipocalin 2 [528–530]. Despite studies in macrophages showing that NRAMP1 can restrict mycobacterial growth, deletion of *Nramp1* in mice infected with *M. tuberculosis* did not result in increased bacterial burden [531]. This is in contrast to *M. avium,* where expression of the *Nramp1*^D169^ allele (which results in deficient function and/or expression) in mice results in these animals being highly susceptible to *M. avium* [532]. *M. avium* growth increased in a dose-dependent manner with iron administration in mice expressing the functional *Nramp1*^*G169*^ allele indicated that excess iron can impair or overcome the function of the NRAMP1 protein [532]. Further studies have argued that NRAMP1 in fact transports iron into the phagosome upon its recruitment to aid mycobacterial killing be catalysing the Fenton/Haber–Weiss reaction to produce hydroxyl radicles [533]. Further investigation is required to fully elucidate the role of NRAMP1 during mycobacterial infections and whether the importance of its role differs depending on the mycobacterial species causing infection.

Murine BMDM infected with *M. avium* upregulated the transcription of heavy-chain (H)-ferritin in response to the stimulation of toll-like receptor-2. This could play a role in starving the mycobacterium of iron by storing iron atoms away from the pathogen while also driving macrophage polarisation towards a glycolytic M1 antibacterial phenotype via HIF1α [534, 535]. Iron deficiency induced stabilisation of the transcription factor HIF-1α promoting sustained IFN-γ-mediated polarization towards the M1 phenotype in vivo and the addition of the iron chelator deferoxamine *to M. tuberculosis*-infected human primary macrophages promoted the M1 phenotype in vitro [535, 536]. LCN2 is involved in the innate immune response and can bind to the bacterial siderophore-iron complex, carboxymycobactin and restricts bacterial growth. In vitro studies showed that Lcn2 restricts the growth of *M. avium *in vitro while the mycobacteria also induced Lcn2 production by murine BMDMs [537]. Further in vivo studies with LCN2, myeloid differentiation primary response (MyD88) and TIR-domain-containing adapter-inducing interferon-β (TRIF) knock out mice confirmed this observation [537]. LCN2 was elevated and reduced *M. avium* growth in the blood of infected mice. Since *M. avium* is an intracellular pathogen, subcellular imaging showed that the mycobacteria avoid LCN2-mediated immunity by residing in the Rab11 + endocytic recycling pathway which still allows them access to transferrin [537]. The Rab5 and Rab7 GTPases were also investigated as they control early and late endosomal fusion, respectively. A well-known strategy that pathogenic mycobacteria employ to persist within macrophages is by inhibiting phagosomal maturation*. *In vitro studies using murine BMDM revealed that those expressing the dominant negative Rab5, Rab5(S34N), limited the concentration of iron within the *M. avium* phagosome which resulted in the phagosome maturing and ultimately killing the mycobacteria when compared to wild-type Rab5 BMDM. Therefore, early endosomal fusion is required for the mycobacteria to persist in these early phagosomal compartments but *M. avium* requires an adequate iron supply to prevent further phagosome maturation [538].

Copper is essential to mycobacteria with its role as a required co-factor in the *aa3*-type cytochrome c oxidase, which is essential for *M. tuberculosis* growth. However, most studies on copper in mycobacteria have focused on resistance to copper stress as copper toxicity in the macrophage phagosome is a known antimycobacterial strategy employed by the host. Several studies have characterised the transcriptional response to copper but the exact mechanisms of copper uptake and not yet fully defined [539]. However, copper uptake is known to be mediated by outer membrane porins in mycobacteria [540]. Other membrane proteins have been identified, such as CtpV which acts as a copper efflux pump and MctB which regulates intracellular copper levels, as being important for *M. tuberculosis* copper resistance and virulence [541, 542]. *M. tuberculosis* senses copper via two copper responsive transcriptional regulators CsoR and RicR [543, 544]. Both of these regulators control genes that contribute to copper resistance and virulence of *M. tuberculosis*. DNA binding of CsoR is affected by binding to copper and derepresses the expression of an operon which includes *ctpV,* in response to high copper levels. RicR regulates a copper induced regulon including the methallothionein *mymT* a copper binding protein that helps to protect against copper toxicity [545]. Mutation of RicR resulting in an inability to respond to copper led to increased copper sensitivity and attenuated virulence in mice suggesting that this regulon is important for protecting *M. tuberculosis* against copper stress during infection [546]. The toxicity of copper in bacteria is thought to be due to copper removing iron from iron sulfur clusters, indeed, transcriptomic data from *M. tuberculosis* under copper stress also implicates damage of iron sulfur cluster enzymes [12, 539].

Zinc is an essential nutrient for mycobacteria and so access to this metal must be crucial for establishing and persisting in the host [547]. Mycobacteria can sense zinc levels and adapt to allow for persistence during zinc limited conditions [548–550]. The zinc import systems in mycobacteria are not clearly defined but are likely to consist of unspecific transport divalent cations transporters from the CorA, MgtE, ZIP, NiCo and Pit families of uptake systems. P-type ATPases may also be able to import zinc ions into the cell [551]. Export of zinc to maintain zinc homeostasis during exposure to high zinc levels is an important resistance mechanism as *M. tuberculosis* infected macrophages import high amounts of zinc into the phagosome. *M. tuberculosis* increases the expression of a P-type ATPase CtpC in response to infection and exposure to zinc. Loss of this ATPase leads to increased zinc levels within the bacteria and a reduced capacity to grow within macrophages [79]. Another gene Rv3929, which is encoded in the same operon as CtpC, is upregulated in response to zinc stress. This gene encodes a putative metallochaperone that could potentially supply zinc to the CtpC transporter. Zinc sequestration may also occur to restrict the growth of extracellular bacteria present in the necrotic granulomas in active TB as S100 proteins are produced at high levels by neutrophils in the granulomas of TB patients[552]. Zinc starvation has been shown to lead to ribosome hibernation in *M. smegmatis* [553]. This ribosome hibernation results in antibiotic tolerance of *M. tuberculosis* in mouse lungs and so while zinc sequestration may restrict the growth of the bacteria it may also potentially cause the bacteria to be more difficult to treat and eradicate during infection [554].

Manganese, nickel and cobalt are also essential nutrients for *M. tuberculosis*. CtpC was also found to transport manganese allowing for metalation of secreted proteins, and deletion of CtpC resulted in altered manganese homeostasis and increased sensitivity to oxidative stress [555]. Nickel is used as a co-factor for the *M. tuberculosis* urease UreA [556, 557]. Mycobacteria are some of the few bacteria that can synthesize vitamin B_12_, a complex cobalt containing molecule, and have three vitamin B_12_-dependent enzymes [558–560]. However, the mechanisms by which these metals are taken up by mycobacteria not yet known. Host sequestration of manganese (and zinc) by S100 proteins, including calprotectin, a chelator of extracellular iron and zinc, is induced in response to bacterial infections [561]. Sequestration of extracellular manganese and zinc is likely to occur via S100 proteins as they are highly produced by neutrophils in the lung granulomas in TB patients [552]. This would likely impact the growth of extracellular bacteria that are present within necrotic granulomas in active TB, but further studies are required to clearly determine the role of these metals in *M. tuberculosis* virulence.

### Therapy

An alteration in metal homeostasis, either systemically or within the lung compartment is associated with the pathogenesis and poor outcomes in several respiratory diseases. Therefore, correcting metal dysregulation could provide a novel approach to treat these diseases, potential counteracting one of the driving forces in the pathogenesis of these diseases. The potential of using iron chelators to reduce pulmonary iron overload in chronic lung diseases such as COPD, CF and IPF are an exciting prospect [319, 562–564]. Reducing iron overload specifically in the lung in chronic lung diseases could help to counteract the metal dysregulation driving chronic inflammation and also potentially limit the respiratory infections that commonly occur in people living with these conditions by depriving airway microbes from this essential nutrient. However, as iron deficiency and anaemia is prevalent in the majority of these diseases, any direct targeting of iron in the lung must avoid reducing systemic iron levels further and re-supplementing systemic iron in iron deficient and anaemic patients may benefit overall survival and exercise capacity. Another approach to counteracting high iron in the lung is the use of gallium, a non-essential metal that acts as an iron mimetic. Gallium containing compounds have been shown to improve the lung function of CF patients with chronic infections. Gallium compounds have been shown to inhibit bacterial iron dependent processes in major respiratory pathogens, *P. aeruginosa* and *M. tuberculosis, M avium, and M. abscessus* [373, 374, 565, 566]. As gallium can replace iron in iron dependent bacterial proteins but is not redox active it interferes with function of iron-dependent essential processes such as the respiratory chain and kills the bacteria. Metal metabolism dysfunction could also potentially be corrected very easily by dietary supplementation, such as selenium supplementation for influenza and zinc supplementation for asthma. However, these treatments are likely to only have a beneficial effect in patients with deficiencies in these metals and so personalised treatment approaches are vital.

Immunonutrition is a novel concept where patients are given a personalized diet rich in vitamins and minerals such as zinc, selenium and iron to modulate immune responses in order to improve disease outcome and recovery. The modulation of the gut microbiota by immunonutrition has been reviewed as a potential therapeutic strategy for obese COVID-19 patients [567]. This novel idea of utilizing the gut-lung axis might also have potential influences on the outcome of bacterial infections such as *M. tuberculosis* that rely on metals such as iron for their replication and growth. Perhaps a personalized diet to deprive the bacteria of certain metals may one day be a potential therapeutic strategy for pulmonary bacterial infections. An overview of potential therapies for respiratory disease by targeting metal homeostasis and dysregulation are presented in Table 2.

**Table 2:** Studies investigating the role of metals in respiratory diseases and potential treatments targeting metal homeostasis/dysregulation

Disease	Metal	Human studies	Therapy	Animal studies
Asthma	Iron	Anaemia is associated with asthma [258] Ferritin stores associated with decreased incidence of asthma [257] Lower non-haem iron in BAL, and increased number of iron-loaded cells seen in asthma patients [261] *DMT1* and *TFR1* expression increased in airway tissue of patients with severe asthma [261]	IV iron administration to correct iron deficiency in asthma patients [567]	Mice: In house dust-mite model, macrophages with highest *Tfr1* also upregulate *Il13* [261] Mice: Iron supplementation decreased airway eosinophilia and type 2 cytokines in ovalbumin-induced allergic asthma model [149] Rats: Dietary iron supplementation causes increased butyrate [264, 568]
	Zinc		Zinc supplementation in children with asthma [569]	
COPD	Iron	Iron metabolism altered in COPD [299–303] Current/former smokers have high iron in sputum, exhaled breath condensate, AMs [211, 304–313] Anaemia and non-anaemic iron deficiency often accompany COPD [314] Anaemia can predict mortality [315–317] Iron-deficient patients have more exacerbations [318] COPD AMs do not suppress hepcidin in response to iron deficiency [317] AMs from smokers have higher iron than non-smokers [310]	Iron chelators to reduce lung iron overload [320, 561–563]	Rats: Iron accumulates in respiratory epithelial cells after smoke exposure smoke [305] Mice: Smoke exposure reduces hepcidin expression in COPD models [54] Mice: Cigarette smoke increases expression of FPN, ferritin and the TFR1 on AMs and inhibits hepcidin induction by LPS [54]
	Zinc	Zinc lower in COPD patients [77, 324] Low dietary zinc in smokers linked to higher incidence of COPD [325] Chronic smokers have increased ZIP8 expression [326] Zinc deficiency allows cadmium to accumulate to toxic levels in smoker’s AMs [332]		Mice: Zinc depletion caused increased lung cadmium burden and permanent lung damage [329] Mice: Zinc deficiency causes increased airway inflammation when exposed to smoke [331] Mice: Zinc supplementation decreases AM numbers after smoke exposure [331]
	Copper	COPD patients with stable disease have lower copper in EBC than healthy non-smokers [336] Patients with Menkes disease have higher incidence of emphysema [337, 338]	Administration of copper/heparin [337]	Copper deficiency induces emphysema in several animal models [333–335] Rats: Copper deficiency reduces AEC integrity [335]
	Selenium	Selenium responsive genes altered in COPD patients [340] Patients with higher selenium have a higher FEV1 [341]		
	Manganese	Patients with severe COPD have higher manganese levels [343]		
Cystic Fibrosis	Iron	Systemic iron deficiency common [359–365] Increased iron in airways and sputum, and within AMs and IMs [366–369, 383] CF macrophages have altered iron metabolism [370] ΔF508-CFTR expressing AECs have altered iron homeostasis, and release more iron [371, 372]	Gallium administration to CF patients with chronic infection to improve lung function [374]	
	Zinc	Serum and plasma zinc are decreased [376, 377] Zinc is elevated in CF airway [383, 384] Regions in the lung enriched with calprotectin allow *S.aureus* to coexist with *P. aeruginosa* [385]		
	Copper	Increased copper in sputum [383] Reduced activity of copper enzymes [386, 387]		
	Selenium	Selenium deficiency is observed [389–392]		
Non-cystic fibrosis bronchiectasis	Iron	Elevated iron in sputum [383, 384]		
	Zinc	Elevated zinc in sputum [383, 384] Zinc may be reduced or unchanged in serum [401, 402]		
Lung Cancer	Iron	Patients have higher ferritin levels [426] Increased LCN2 and TFR seen [427, 428] Increased miR-20a expression in NSCLC inhibits ferroportin expression [431]	Iron deprivation, iron supplementation to supress tumours [425] Anti-transferrin therapy [425]	Mice: Downregulation of LCN2 or TFR in adenocarcinoma model suppressed tumour growth [427, 428] Mice: Iron given *i.v.* inhibited tumour growth [437]
	Zinc	Decreased serum zinc is a biomarker of lung cancer [422] Increased plasma zinc correspond with lower risk of lung cancer [424]		
	Copper	Increased copper in serum [422]		
	Manganese	Increased MnSOD activity [438–440] Polymorphisms in MnSOD linked to higher risk of lung cancer [441]		
IPF	Iron	Patients have increased iron deposition [459, 460] Iron is elevated in BAL fluid [458] BAL cells have increased iron-dependent ROS generation [462]	Iron chelators to reduce lung iron overload [320, 561–563]	
	Zinc	Reduced zinc in BAL fluid [458]		Mice: Cu,Zn-SOD/SOD1 deficient mice are protected from asbestos induced lung injury [470]
	Copper	Copper is elevated in BAL fluid [458] LOXL2 expression is increased [467]		Mice: LOXL2 expression increased by bleomycin [466] Mice: LOXL2 inhibitory antibody protects against bleomycin induced lung injury [468]
	Other	Chromium, nickel, manganese lower in BAL fluid [458]		
PAH	Iron	Iron deficiency and/or anaemia common in PH patients [478, 479] PH is a major cause of mortality in those with chronic haemolytic anaemias [480] Iron accumulation in lungs and AMs of PH patients [482] 70% of those with G208C mutation in NFU1 develop PAH [481]	Iron supplementation in iron deficient patients [322]	Mice: Loss of IRP1 leads to PH [485] Mice: Expression of hepcidin-resistant FPN in smooth muscle causes PAH [483]
	Other	Zinc and copper transport implicated in pulmonary vascular homeostasis [487, 488] Selenium may be elevated in PH [489]		Mice: Low copper diet in PH model has no effect on RV failure [490] Chicken: Dietary supplementation of selenium in PH model prevented RV hypertrophy [491]
Mycobacterial infections (TB & NTM)	Iron		Gallium compounds to inhibit bacterial iron dependent proteins and siderophores [375, 564, 565] Modulation of iron to support host TB defences [7, 534] Pyrazolopyrimidinone (PZP)—anti mycobacterial compound chelates intracellular iron [570] Synthetic fluorescent iron chelators restrict *M.avium* within macrophages [571]	Mice: Infected macrophages take up iron and deliver it to phagosomes containing mycobacteria via TFR1 [83] Mice: *Nramp1*^D169^ (functionally deficient) expression increases susceptibility to *M. avium* infection in mice [531] Mice: Expression of functional NRAMP1 had *M. avium* growth that increased with iron administration [531] Mice: BMDMs infected with *M. avium* upregulate H-ferritin[533] Mice: *M. avium* infection induced LCN2, which in turn can limit *M. avium* growth [536]
	Zinc	Zinc is essential for mycobacteria [546] Neutrophils in necrotic granulomas produce S100 proteins, possibly to sequester zinc and inhibit bacterial growth [551] Zinc deficiency in *M. smegmatis* causes ribosomal hibernation [552]		Mice: Zinc starvation causing ribosome hibernation in *M. tuberculosis* may lead to antibiotic tolerance [553]
	Copper	Copper responsive transcription factors control genes important for *M. tuberculosis* virulence [542, 543]		Mice: RicR mutation reduced *M. tuberculosis* virulence [545]
	Other	Manganese, nickel and cobalt are essential nutrients for *M. tuberculosis* [554]		

## Conclusion

The lungs are a unique compartment in the body in that they are constantly exposed to the environment. We are constantly breathing in air from our surroundings which consists of microorganisms, particles, chemicals, and pollutants. The resident immune cells within this niche must be able to deal with the influx of microbes and particles without causing inflammation. Unfortunately, environmental, lifestyle and genetic factors can lead to chronic lung inflammation resulting in an array of lung diseases that result in obstruction and damage to the airways and a decline in lung function. Furthermore, recurrent respiratory infections are common among those with chronic lung disease and contribute to progressive decline in lung function. The role of metabolites and nutrients present in lung microenvironment are just beginning to be investigated and appreciated. Metals essential to most organisms, such as iron, copper and zinc are found to be altered in several lung diseases. These metals play important roles both in the function of immune cells in the lung and in the microbes present in the airways. There remains much to be explored on the cause and source of this metal dysregulation during lung disease, as well as the exact mechanistic consequences of this on the immune cells and the constituents of the airway microbiota. We believe that targeting disturbed metal metabolism seen in many lung diseases is a novel approach that could potentially address both immune-driven pathology and the increased occurrence of respiratory infections. We hope that future work will further our understanding of the complex interactions between lung immunity, the lung microbiome and the lung microenvironment and will lead to much improved treatments for the many diseases of the lung that yet remain to be curable.

## Data Availability

Not applicable.

## Abbreviations

2DG: 2-Deoxy-d-glucose
AD: Adenocarcinoma
AEC: Alveolar epithelial cells
ALAS: 5-Aminoevulinate synthase
AM: Alveolar macrophage
ATP: Adenosine triphosphate
BALF: Bronchoalveolar lavage fluid
BCR: B cell receptor
Bfr: Bacterioferritin
BMDM: Bone marrow derived macrophage
cDCs: Conventional dendritic cells
CF: Cystic fibrosis
CFTR: CF transmembrane conductance regulator
COPD: Chronic obstructive pulmonary disease
CXCR: Chemokine receptor
DAMP: Damage associated molecular patterns
DCs: Dendritic cells
DMT1: Divalent metal transporter 1
EBC: Exhaled breath condensate
EPX: Eosinophil peroxidase
FEV1: Forced expiratory volume in 1s
FPN: Ferroportin
FTH: Ferritin heavy chain
GM-CSF: Granulocyte macrophage-colony stimulating factor
Gpx4: Glutathione peroxidase 4
HFE: Hereditary hemochromatosis (HH) susceptibility gene
HIF-1α: Hypoxia inducible factor-1α
HO-1: Heme oxygenase 1
iBALT: Bronchial associated lymphoid tissue
IFN-ɣ: Interferon-ɣ
ILCs: Innate leukocyte cells
IM: Interstitial macrophage
iNKT: Invariant natural killer cells
IPF: Idiopathic pulmonary fibrosis
IPL: Isolated perfused lungs
IRF: Interferon regulatory factor
iTAMs: Iron-loaded TAMs
JNK: C-Jun N-terminal kinase
KIR: Killer cell-immunoglobulin like receptor
LCN-2: Lipocalin-2
LFA: Lymphocyte function-associated antigen
LfR: Lactoferrin receptor
LIAS: Lipoic acid synthase
LOXL2: Lysyl oxidase-like 2
LPS: Lipopolysaccharide
LRP1: Lipoprotein receptor related protein 1
LTi: Lymphoid tissue inducer
MAIT: MR1-restricted T cells
MHC: Major histocompatibility complex
MnSOD: Manganese superoxide dismutase
moDCs: Monocyte-derived dendritic cells
MPO: Myeloperoxidase
mRNA: Messenger ribonucleic acid
MsrB1: Methionine sulfoxide reductase B1
MTs: Metallothioneins
MyD88: Myeloid differentiation primary response
NETs: Neutrophil extracellular traps
NK: Natural killer
NO: Nitric oxide
NRAMP1: Natural resistance associated macrophage protein 1
NTM: Non-tuberculous mycobacteria
OH·: Hydroxyl radical
OX PHOS: Oxidative phosphorylation
PAH: Pulmonary arterial hypertension
pDCs: Plasmacytoid DCs
PDH: Pyruvate dehydrogenase
PH: Pulmonary hypertension
PHDs: Prolyl hydroxylases
RNSO: S-nitrosothiols
ROS: Reactive oxygen species
SC: Squamous cell
SCD: Sickle cell disease
SCFA: Short chain fatty acid
SCLC: Small cell lung cancer
SelK: Selenoprotein K
SOD: Superoxide dismutase
STAT: Signal transducer and activator of transcription
TAMs: Tumour associated macrophages
TB: Tuberculosis
TCR: T cell receptor
TGF: Transforming growth factor
Th: Helper T cell
TLR: Toll like receptor
TLS: Tertiary lymphoid structures
TME: Tumour microenvironment
Tregs: T regulatory cells
TFR1: Transferrin receptor 1
TRIF: TIR-domain-containing adapter-inducing interferon-β
T_RM_: Lung resident memory T cells
tRNA: Transfer ribonucleic acid
αKGDH: α-Ketoglurate dehydrogenase
β2-m: β2-Microglobulin
